# Prevalence of Use of Potentially Inappropriate Medications Among Older Adults Worldwide: A Systematic Review and Meta-Analysis

**DOI:** 10.1001/jamanetworkopen.2023.26910

**Published:** 2023-08-02

**Authors:** Fangyuan Tian, Zhaoyan Chen, Ya Zeng, Qiyi Feng, Xi Chen

**Affiliations:** 1 Department of Pharmacy, National Clinical Research Center for Geriatrics, West China Hospital, Sichuan University, Chengdu, China; 2 Department of Epidemiology and Health Statistics, West China School of Public Health and West China Fourth Hospital, Sichuan University, Chengdu, China; 3 Precision Medicine Research Center, Sichuan Provincial Key Laboratory of Precision Medicine and National Clinical Research Center for Geriatrics, West China Hospital, Sichuan University, Chengdu, China; 4 Department of Integrated Care Management Center, West China Hospital, Sichuan University, Chengdu, China

## Abstract

**Importance:**

The use of potentially inappropriate medications (PIMs) is widespread yet continues to receive little attention in outpatient services.

**Objective:**

To estimate the overall prevalence of PIM use in outpatient services.

**Data Sources:**

PubMed, Embase, and Web of Science were searched to identify relevant studies published from January 1, 1990, to November 21, 2022.

**Study Selection:**

Observational studies that reported the prevalence of PIM use among older patients in outpatient services were screened.

**Data Extraction and Synthesis:**

Two reviewers independently selected eligible articles, extracted data, and assessed the risk of bias. A random-effects meta-analysis was conducted to pool the prevalence estimates.

**Main Outcomes and Measures:**

The global patterns in the prevalence of PIM use among older patients in outpatient services were estimated, and the temporal trends and regional differences in PIM use were investigated.

**Results:**

A total of 94 articles with 132 prevalence estimates were analyzed, including nearly 371.2 million older participants from 17 countries. Overall, the pooled prevalence of PIM use was 36.7% (95% CI, 33.4%-40.0%). Africa had the highest prevalence of PIM use (47.0%; 95% CI, 34.7%-59.4%), followed by South America (46.9%; 95% CI, 35.1%-58.9%), Asia (37.2%; 95% CI, 32.4%-42.2%), Europe (35.0%; 95% CI, 28.5%-41.8%), North America (29.0%; 95% CI, 22.1%-36.3%), and Oceania (23.6%; 95% CI, 18.8%-28.8%). In addition, the prevalence of PIM use is highest in low-income areas. Use of PIMs among older patients has become increasingly prevalent in the past 2 decades.

**Conclusions and Relevance:**

This study of patterns of PIM use by different groups, such as geographic regions and World Bank countries, suggests noticeable geographic environment and economic income differences in the burden of PIMs in outpatient services. Furthermore, the high prevalence trend in the past 2 decades indicates that the global burden of PIM use continues to be worthy of attention.

## Key Points

**Question:**

What is the prevalence of use of potentially inappropriate medications (PIMs) among older patients in outpatient services?

**Findings:**

In this systematic review and meta-analysis, 94 articles with 132 prevalence estimates were analyzed, including nearly 371.2 million older participants from 17 countries. Overall, PIMs had a pooled prevalence of 36.7%, and PIM use among older patients has become increasingly prevalent in the past 2 decades.

**Meaning:**

These results suggest an increasing risk of PIM use in older outpatients, highlighting the need for worldwide health care reforms and improvements in drug safety for outpatients.

## Teaser

This systematic review with meta-analysis examines the worldwide prevalence of potentially inappropriate medication use among older outpatients.

## Introduction

With the extension of life expectancy and the reduction of fertility, population aging is an inevitable result; aging and negative population growth are also mutually reinforcing processes.^1^ It is estimated that the number of people 65 years or older was 727 million in 2020, and this number is expected to increase to 1.5 billion by 2050.^2^ With the continuous increase in the number and proportion of the older population, the degree of aging in the world will continue to deepen. Population aging is not only a focus of social attention but also a major health issue facing this era. As the body gradually ages, weakness is inevitable, and older individuals become prone to multimorbidity, especially chronic diseases. Currently, the treatment of chronic diseases in older individuals tends to focus on a single disease. With the increase in combined chronic diseases, older patients often have to use multiple medications at the same time. The use 5 or more medications by a patient at the same time is called polypharmacy.^3^

Among various drugs used by older patients, the potential adverse risks of a certain medication may exceed the expected benefits; such drugs are referred to as potentially inappropriate medications (PIMs).^4^ The Beers criteria were the first expert consensus on PIMs in the geriatric population^5^; the American Geriatrics Society, through an expert US-based panel, has undertaken the task of regular review and updating of the Beers criteria, which are now in their sixth iteration. University College Cork organized experts from many disciplines to formulate the Screening Tool of Older People’s Prescriptions/Screening Tool to Alert to Right Treatment (STOPP/START) criteria through the Delphi method, and the second edition was updated in 2014.^6^ These 2 criteria have been widely used worldwide.

According to the results of previous studies, PIM use in older patients is closely related to an increase in the incidence of adverse drug events (ADEs) and emergency department visits.^7,8^ Meanwhile, compared with patients without PIM use, their health-related quality of life is worse. The use of PIMs by older outpatients will increase outpatient drug fees and outpatient visits, thereby increasing the use of outpatient medical resources and increasing the risk of hospitalization for older outpatients.^9,10^ Outpatient services are the medical services that most patients receive on their first visit to the hospital. The outpatient base is larger than the inpatient base, and the range of medications is more complex than that of patients in primary care. Therefore, the safety of medication use in outpatient services is of great importance. Currently, there have been meta-analyses^11,12^ of PIMs in primary care settings, but uncertainties remain about the context of PIM use in outpatient services. One study^11^ reported a decrease in the prevalence of PIM use. However, another study^12^ showed that the prevalence of PIM use increased from 31.94% in 2016 to 42.67% in 2018 in outpatient services. In the current study, we sought to conduct a systematic review with meta-analysis to estimate the overall prevalence of PIM use among older patients in outpatient services.

## Methods

### Eligibility Criteria

The inclusion and exclusion criteria for this systematic review and meta-analysis were as follows: (1) recruited older (aged ≥60 years or aged ≥65 years) outpatients (patients who visited outpatient departments), (2) reported point prevalence (number of older patients with PIM use during the study period divided by the total number of older patients during the study period) of PIM use (determine on explicit PIM criteria), and (3) provided statistical information or raw data on the outcomes. Studies were excluded if they (1) recruited participants not from outpatient services, such as those from nursing homes; (2) did not assess PIM use based on published explicit PIM criteria; and (3) had only a PIM article with a country. The Preferred Reporting Items for Systematic Review and Meta-analysis (PRISMA) checklist was followed in this study.^13^

### Search Strategy and Literature Screening

The literature was retrieved from PubMed, Embase, and Web of Science from January 1, 1990, to November 21, 2022. We used the following search terms: *outpatient service*, *older*, and *potentially inappropriate medication*. All search strategies were developed and implemented independently by 2 investigators (Z.C. and Y.Z.) and then cross-checked. A sample of the search strategy is given in eAppendix 1 in Supplement 1. Reference lists of identified studies were also searched to avoid missing potentially relevant studies. After using EndNote X7 to screen duplicates, the 2 researchers read the title and abstract of these articles back to back for preliminary screening and further read the full text that initially met the inclusion criteria to determine whether they were finally included. When the 2 researchers disagreed, a third researcher (F.T.) made the final decision. The protocol was registered with PROSPERO (CRD42022377692).

### Data Extraction

The following data were extracted from each included study: (1) the basic characteristics of the study, including the first author, publication year, country, location, study design, sample period, age of definition of older, sample size, mean number of drugs (polypharmacy defined as >5 drugs), proportion of male patients, and population; (2) PIM criteria, number of patients using 1 or more PIMs, and prevalence of PIM use; and (3) quality of the study as assessed by the methods of Hoy et al.^14^

### Statistical Analysis

Meta-analysis was performed by using R software, version 4.2.0 (R Foundation for Statistical Computing). All resistance data collected were normalized using the arcsine transformation, and the normality of untransformed and transformed data was verified using the Shapiro-Wilk method. We used anticipated heterogeneity and the *I*^2^ statistic to assess statistical heterogeneity. When there was statistical heterogeneity, owing to the anticipated heterogeneity of the included studies, we used a random-effects model to estimate effect sizes, which would provide more conservative estimates of the 95% CIs.^15^ Subgroup analysis was performed for different collection geographic regions, World Bank countries, periods, PIM criteria, locations, study designs, mean ages, mean numbers of drugs, percentages of males, populations, and sample sizes. The pooled result is presented as the mean prevalence with a 95% CI. Sensitivity analyses were conducted by omitting 1 study at a time and recalculating the pooled prevalence for the remaining studies to identify studies with outlying prevalence estimates that may bias the pooled result. A 2-sided *P* < .05 was considered statistically significant. We performed a publication bias test using a funnel plot and the Egger test.^16^

## Results

### Study Selection

**Figure 1.**
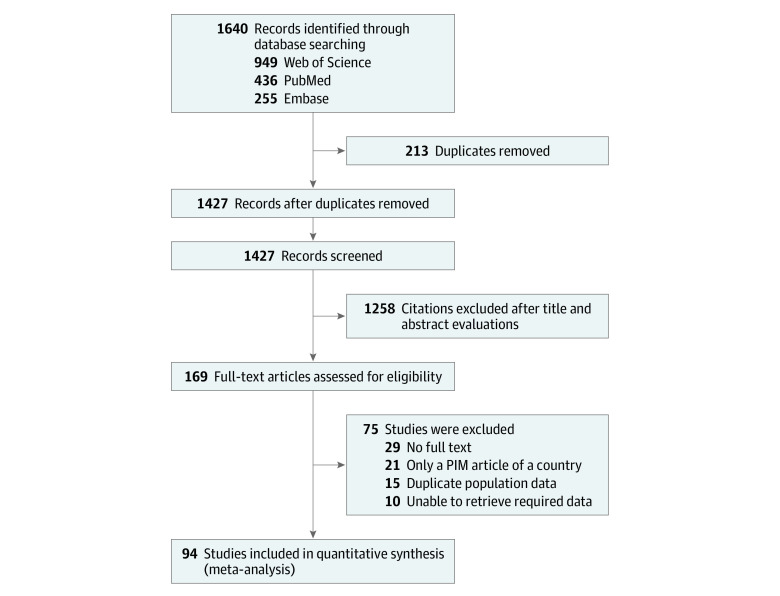
Flowchart of the Literature Search PIM indicates potentially inappropriate medication.

A total of 1640 studies were identified, including 436 studies from PubMed, 255 studies from Embase, and 949 studies from Web of Science. Following exclusion of 213 duplicate studies, 1258 studies were excluded by the titles and abstracts. Ninety-four studies^11,17,18,19,20,21,22,23,24,25,26,27,28,29,30,31,32,33,34,35,36,37,38,39,40,41,42,43,44,45,46,47,48,49,50,51,52,53,54,55,56,57,58,59,60,61,62,63,64,65,66,67,68,69,70,71,72,73,74,75,76,77,78,79,80,81,82,83,84,85,86,87,88,89,90,91,92,93,94,95,96,97,98,99,100,101,102,103,104,105,106,107,108,109^ with 371 224 692 older outpatients were included for analysis (Figure 1; eAppendix 2 in Supplement 1).

### Study Characteristics

The 94 studies were conducted across 17 countries. Forty-one studies^17,18,19,20,21,22,23,24,25,26,27,28,29,30,31,32,33,34,35,36,37,38,39,40,41,42,43,44,45,46,47,48,49,50,51,52,53,54,55,56,57^ were conducted in Asia, followed by 22 studies^11,58,59,60,61,62,63,64,65,66,67,68,69,70,71,72,73,74,75,76,77,78^ in Europe, 14 studies^79,80,81,82,83,84,85,86,87,88,89,90,91,92^ in North America, 8 studies^93,94,95,96,97,98,99,100^ in South America, 7 studies^101,102,103,104,105,106,107^ in Africa, and 2 studies^108,109^ in Oceania. Between 1999 and 2021, a total of 51 studies^11,37,38,39,40,41,42,49,50,51,52,53,54,55,56,57,58,59,60,61,62,63,64,65,66,67,68,69,70,71,72,73,74,75,76,79,80,81,82,83,84,85,86,87,88,89,90,91,92,108,109^ were performed in high-income countries, 23 studies^17,18,19,20,21,22,23,24,25,26,27,28,29,77,78,93,94,95,96,97,98,99,100^ in upper- to middle-income countries, 17 studies^30,31,32,33,34,35,36,43,44,45,46,47,48,101,102,103,104^ in lower- to middle-income countries, and 3 studies^105,106,107^ in low-income countries. Sixty-three studies^17,20,21,22,23,24,26,27,28,29,30,31,33,34,35,43,47,48,49,50,52,53,54,55,56,57,58,60,61,62,64,69,73,75,79,80,81,82,83,84,85,86,87,90,91,92,93,94,95,96,97,99,100,101,102,103,104,105,106,107,109^ of the PIM prevalence estimates were based on the Beers criteria, 26 studies^17,19,25,26,32,34,36,40,43,44,45,46,48,51,60,65,66,72,74,76,77,78,89,96,101,107^ were based on the STOPP/START criteria, 13 studies^17,18,20,27,28,37,38,39,41,42,50,64,65^ on localization criteria that were developed based on the characteristics of medication for older patients in their own country, and 13 studies^11,50,59,63,67,68,70,71,79,85,88,98,108^ on other criteria. Eighty-five studies^11,17,18,19,20,21,22,23,24,25,26,27,28,29,30,31,32,33,34,35,36,37,38,39,40,41,42,43,44,45,46,47,48,49,50,51,52,53,54,55,56,57,58,59,60,64,65,66,67,68,69,70,73,74,75,76,77,79,81,82,83,84,86,87,88,89,90,91,92,93,94,95,96,97,98,99,101,102,103,104,105,106,107,108,109^ were cross-sectional in design, and 11 studies^11,29,41,49,50,51,71,80,88,90,91^ were conducted in a national population survey. The mean age of the participants in most studies (65 studies^17,18,19,21,22,23,25,27,30,31,32,33,36,37,38,40,42,43,44,45,46,47,48,49,51,52,53,54,55,56,58,59,61,62,64,67,68,71,72,74,75,76,77,80,81,83,84,85,86,87,88,89,92,93,94,97,98,100,101,102,103,104,105,107,108,109^) was younger than 80 years and older than 65 years, and 31 studies^18,25,27,30,33,36,38,43,44,46,47,48,49,53,55,58,59,60,66,69,71,72,74,77,83,84,85,93,98,100,101^ had a mean number of drugs greater than 5. Thirty-four studies had a male ratio of more than 50%, and 34 studies^17,22,24,25,29,33,34,38,42,45,47,48,49,51,53,55,57,65,66,71,72,73,74,75,85,87,90,92,94,96,99,100,108,109^ included older outpatients with certain diseases. Twenty-six studies^11,19,22,23,27,28,29,37,41,50,51,52,54,59,61,62,63,64,68,70,73,79,80,88,90,91^ had a sample size of more than 10 000 people (Table; eAppendix 2 in Supplement 1).

**Table.** Stratified Meta-Analysis of the Prevalence of PIM Use

Characteristic	No. of studies (No. of data points)	Pooled prevalence of PIM use (95% CI)	*I*^2^, %	*P* value
Geographic region				
Asia	41 (56)	0.37 (0.32-0.42)	100	<.001
Europe	22 (39)	0.35 (0.28-0.42)	100	<.001
North America	14 (16)	0.29 (0.22-0.36)	100	<.001
South America	8 (10)	0.47 (0.35-0.59)	98.03	<.001
Africa	7 (9)	0.47 (0.35-0.59)	97.21	<.001
Oceania	2 (2)	0.24 (0.19-0.29)	74.86	.05
World Bank country				
High	51 (73)	0.33 (0.29-0.38)	100	<.001
Upper-middle	23 (34)	0.40 (0.34-0.45)	99.89	<.001
Lower-middle	17 (21)	0.41 (0.33-0.48)	97.87	<.001
Low	3 (4)	0.56 (0.37-0.75)	97.30	<.001
Periods				
≤2000	4 (6)	0.24 (0.16-0.33)	99.87	<.001
2001-2005	10 (10)	0.24 (0.15-0.35)	100	<.001
2006-2010	16 (23)	0.30 (0.23-0.37)	100	<.001
2011-2015	20 (29)	0.39 (0.33-0.46)	100	<.001
2016-2020	48 (64)	0.42 (0.37-0.46)	100	<.001
PIM criteria				
Beers criteria 1997	2 (2)	0.15 (0.06-0.28)	99.96	<.001
Beers criteria 2003	15 (15)	0.26 (0.20-0.33)	100	<.001
Beers criteria 2012	9 (9)	0.33 (0.23-0.43)	99.84	<.001
Beers criteria 2015	19 (24)	0.40 (0.32-0.48)	99.98	<.001
Beers criteria 2019	18 (18)	0.46 (0.38-0.54)	99.44	<.001
STOPP/START V1	4 (4)	0.48 (0.38-0.58)	88.45	<.001
STOPP/START V2	22 (22)	0.39 (0.28-0.50)	99.93	<.001
Localization criteria	12 (15)	0.36 (0.29-0.44)	100	<.001
Others	13 (23)	0.34 (0.27-0.41)	100	<.001
Location				
Regional	80 (111)	0.37 (0.34-0.41)	99.99	<.001
National	11 (18)	0.34 (0.24-0.43)	100	<.001
NR	3 (3)	0.34 (0.19-0.50)	98.66	<.001
Study design				
Cross-sectional study	85 (122)	0.37 (0.33-0.40)	100	<.001
Cohort study	7 (8)	0.30 (0.20-0.41)	100	<.001
Others	2 (2)	0.71 (0.10-1.00)	99.41	<.001
Mean age, y				
>65-80	65 (83)	0.37 (0.33-0.41)	100	<.001
≥80	9 (16)	0.42 (0.33-0.51)	99.99	<.001
NR	20 (33)	0.33 (0.27-0.40)	100	<.001
Mean No. of drugs				
<5	21 (31)	0.35 (0.29-0.42)	100	<.001
≥5	31 (45)	0.46 (0.40-0.52)	99.94	<.001
NR	42 (56)	0.30 (0.26-0.35)	100	<.001
Male, %				
<50	52 (77)	0.35 (0.32-0.39)	100	<.001
≥50	34 (43)	0.41 (0.34-0.48)	99.99	<.001
NR	8 (12)	0.30 (0.19-0.42)	100	<.001
Population				
Outpatients	60 (87)	0.37 (0.33-0.40)	100	<.001
Outpatients with certain diseases	34 (45)	0.37 (0.30-0.43)	100	<.001
Sample size				
<1000	53 (62)	0.40 (0.36-0.45)	97.86	<.001
1000-9999	15 (22)	0.46 (0.39-0.54)	99.78	<.001
≥10 000	26 (48)	0.31 (0.24-0.33)	100	<.001
Overall	94 (132)	0.37 (0.33-0.40)	100	<.001

Abbreviations: NR, not reported; PIM, potentially inappropriate medication; STOPP/START, Screening Tool of Older People’s Prescriptions/Screening Tool to Alert to Right Treatment; V1, version 1; V2, version 2.

### Global and Regional Prevalence of PIM Use

**Figure 2.**
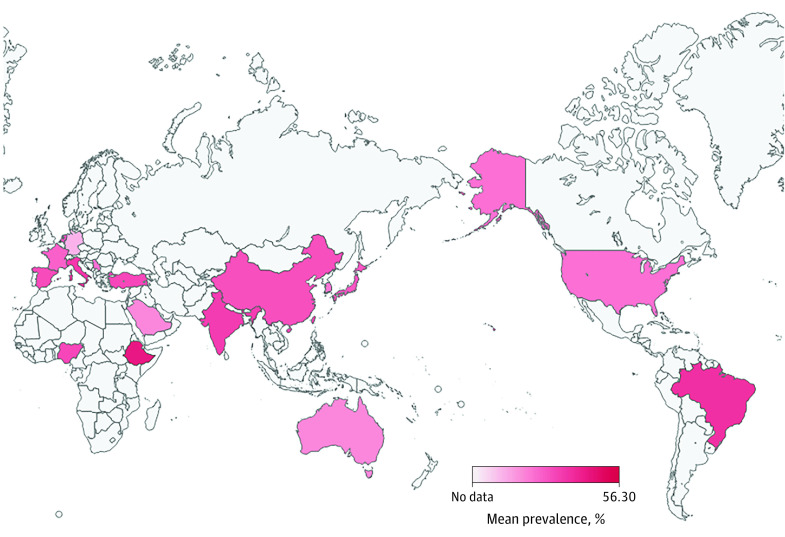
Mean Prevalence Map of Potentially Inappropriate Medication Use in Outpatient Services

The prevalence of PIM use among older outpatients ranged from 1.3% to 95.2% in the studies, and the most commonly used PIMs were benzodiazepines (eAppendix 7 in Supplement 1). The prevalence distribution map of PIM use worldwide in outpatient services ranged from 14% to 56.3% (Figure 2; eAppendix 4 in Supplement 1). In the meta-analysis, the pooled prevalence of PIM use was estimated at 36.7% (95% CI, 33.4%-40.0%) across all included studies (eAppendixes 2 and 5 in Supplement 1). The mean prevalence of PIM use was 47.0% (95% CI, 34.7%-59.4%) in Africa, followed by South America (46.9%; 95% CI, 35.1%-58.9%), Asia (37.2%; 95% CI, 32.4%-42.2%), Europe (35.0%; 95% CI, 28.5%-41.8%), North America (29.0%; 95% CI, 22.1%-36.3%), and Oceania (23.6%; 95% CI, 18.8%-28.8%). The quality assessment of studies is presented in eAppendix 3 in Supplement 1. In the sensitivity analysis, the pooled prevalence ranged from 36.2% to 37.1% when we omitted studies one at a time (the forest plot of leave-one-out analyses is shown in eAppendix 8 in Supplement 1). There was also no publication bias in the Egger test or based on visual inspection of the funnel plot (eAppendix 9 in Supplement 1).

### Subgroup Analysis

**Figure 3.**
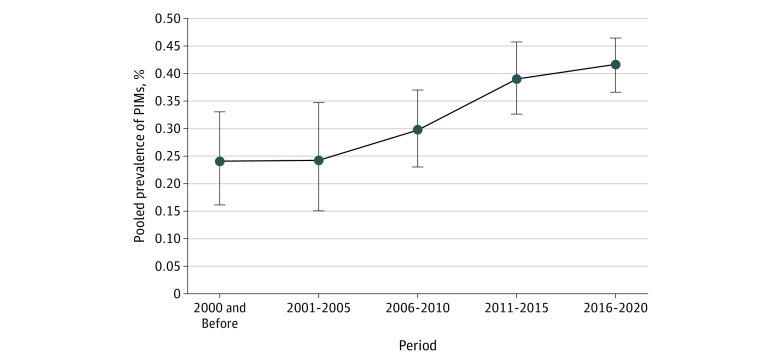
Change in Time of the Prevalence of Potentially Inappropriate Medication (PIM) Use in Outpatient Services Error bars indicate 95% CIs.

The subgroup analysis of the prevalence of PIM use by different geographic regions, World Bank countries, periods, PIM criteria, location, study design, mean age, mean number of drugs, percentage of male participants, population, and sample size is given in the Table. On the basis of the World Bank country of the studies, the estimated pooled prevalence of PIM use was 33.2% in high-income countries, which is the lowest of all countries, 39.5% in upper- to middle-income countries, 40.8% in lower- to middle-income countries, and 56.3% in low-income countries. For example, France, the US, and Australia, which are high-income countries, demonstrated relatively lower pooled prevalence, whereas countries such as China, India, and Brazil, which are middle-income countries, had relatively lower pooled prevalence, and low-income countries, such as Ethiopia, had the highest pooled prevalence. The global prevalence of PIM use in outpatient services, especially in high-income countries, has changed over time worldwide during the past 20 years, displaying an increasing trend (Figure 3; eAppendix 6 in Supplement 1). Of all the Beers criteria, the criteria from 2019 were the most sensitive (46.0%; 95% CI, 38.1%-54.0%). The pooled prevalence of PIM use was higher among polypharmacy participants (45.9%; 95% CI, 40.5%-51.5%) 80 years or older (41.9%; 95% CI, 33.3%-50.9%). There was a difference in the prevalence of PIM use by study design, location, percentage of males, population, and sample size among the studies.

## Discussion

To the best of our knowledge, this is the first meta-analysis to assess the global prevalence of PIM use among older patients in outpatient services. This study analyzed data from 94 studies with 132 prevalence estimates that involved nearly 371.2 million people from 17 countries, providing an up-to-date global PIM prevalence of 36.7% (95% CI, 33.4%-40.0%) that extends to 2022. A previous meta-analysis^110^ with studies until 2019 found a prevalence of PIM use of 33.3% among older persons in primary care settings. In contrast, our study has an updated timeline and includes a wider population. Compared with the previous meta-analysis^110^ of studies in primary care settings, we found a higher prevalence of PIM use in outpatient services. This finding may be mainly due to the higher prevalence of PIM use among older patients in outpatient settings because of more complex diseases and more types of medication compared with primary care settings. From the results obtained in the study, benzodiazepines were the most common PIMs. The high prescription rate of benzodiazepines could be attributed to the high prevalence of insomnia in the geriatric population. Because of the gradual aging of the functions of various organs and tissues, the geriatric population is more sensitive to drugs, and although benzodiazepines are thought to be associated with an increased risk of cognitive impairment, falls, and fractures, they are still common in the geriatric population.^111^ Therefore, almost all criteria list this type of medication as potentially inappropriate.

The subgroup analysis by geographic region showed significant differences in the pooled prevalence of PIM use. Our analysis showed that the prevalence of PIM use was highest in Africa, followed by South America. Medical conditions in Africa are relatively suboptimal, and the fact that almost every other region has PIM criteria for older people in their own region, which is rarely seen in Africa, may contribute to the highest prevalence of PIM use in this region. This result is consistent with a meta-analysis^112^ that found the pooled proportion of multimorbidity in South America. Some studies^17,113^ have found that with the increase in multimorbidity in older outpatients, the risk of PIM use gradually increases. Therefore, there is a certain correlation between multimorbidity and PIMs, and the prevention of chronic diseases is of great significance for improving drug safety in older populations. The prevalence of PIM use is roughly opposite to the national economic level. The prevalence of PIM use is higher in countries with poor economic conditions, and the opposite is true in countries with good economic conditions. When economic conditions are better, the medical environment is usually better, and medical insurance is more perfect, which makes the rational use of drugs more strictly controlled. In addition, the establishment of geriatric medicine has a positive effect on reducing the prevalence of PIM drugs in older patients. Currently, most studies mainly evaluate PIM use through PIM criteria. These criteria are mainly divided into 3 categories: explicit criteria, implicit criteria, and mixed criteria.^114^ Among them, explicit criteria account for the majority and are formulated based on evidence-based guidance and data on irrational drug use.^6,115,116^ All the original studies included in our study were evaluated using explicit criteria. The most used criteria were the Beers criteria, followed by the STOPP/START criteria. The Beers criteria of 2019 were the most sensitive, and with the update of the Beers criteria, the prevalence of PIM use was increasing. This finding is mainly due to the continuous addition of some PIM entries to the Beers criteria update. Some localization criteria developed based on populations in their own countries do not exhibit better advantages compared with the Beers criteria and STOPP/START criteria, which may be because of the relatively small number of relevant studies included in our analysis. This result still needs to be carefully explained.

Patients older than 80 years and polypharmacy patients were more likely to receive PIMs. The oldest patients generally have worse health and more multimorbidity than the general population of older adults, and they are more likely to be exposed to PIM use.^117^ In a previous meta-analysis^118^ of polypharmacy and PIM prevalence in older patients in China, polypharmacy was found to be an independent risk factor for PIM use. Each time a medication in the PIM criteria is added, the PIM exposure risk increases by 5.2%.^119^ There was a difference in the prevalence of PIM use by study design, location, percentage of males, population, and sample size among the studies. This disparity in PIM prevalence could be due to study designs with varying levels of methodologic differences, such as various study populations, sampling procedures, sample coverage, sample sizes, data collection, and so on.

Calculating the global prevalence of PIM use based on the 5-year interval between publications of the study is one of the most important findings of our study. On the basis of our findings, the prevalence of PIM use has changed considerably during the past 2 decades. This finding illustrates the increasing global burden of PIM use. During the past decade, the global prevalence of PIM use among older outpatients has exceeded 40%, indicating a high burden of PIM use for many years. The proportion of the world’s older population increased in the past 20 years; the older population is a group that is susceptible to multiple diseases due to aging, especially chronic diseases. This susceptibility has led to an increase in PIM use in older patients during outpatient visits. For example, studies have shown that prescriptions of antidepressants in outpatient settings have increased nearly twice, from 5.2% in 2002 to 10.1% in 2012,^120^ whereas antidepressants are listed as PIMs in most criteria. Reducing PIMs can reduce ADEs in older patients, especially level 3 ADEs that may cause or contribute to short-term admission.^115^

### Limitations

This study has several limitations that need attention. First, few studies in low-income countries were included in the meta-analysis, which may lead to certain differences in research results reflecting the true prevalence of PIM use in low-income countries. Second, some countries have relatively few studies to include, which may lead to high or low prevalence of PIM use in some countries, and changes in PIM criteria may lead to an increased prevalence of PIM use. Third, the data we analyzed were about PIM use in older outpatients because outpatients are rarely followed up. The effect of PIM use on older patients’ long-term health outcomes is unclear.

## Conclusion

This systematic review and meta-analysis summarizes the prevalence of PIM use in outpatient services. Overall, PIM use was found to occur among nearly 37% of older outpatients, with an increasing trend in the last 2 decades and an even higher prevalence in some low-income countries. The high prevalence of PIM use highlights the global need for health care reforms and improvements in drug safety in outpatient settings.

## Notes

Supplement 1. **eAppendix 1.** A Sample of the Search Strategy **eAppendix 2.** Characteristics of Included Studies **eAppendix 3.** Risk of Bias Assessment **eAppendix 4.** Meta-Analysis of the Prevalence of PIMs in Outpatients Ordered by Countries **eAppendix 5.** Forest Plot of the Overall Prevalence of PIM in Outpatient Services **eAppendix 6.** Stratified Meta-Analysis of the Trend of the Prevalence of PIM for Each Group in World Bank Countries **eAppendix 7.** The List of the Top 10 Most Common Drugs Used With PIM Criteria **eAppendix 8.** Leave-One-Out Sensitivity Analyses **eAppendix 9.** Funnel Plot for the Prevalence of PIMs
eReferences

Supplement 2.
Data Sharing Statement
