# Supplementary material for: Prevalence of Use of Potentially Inappropriate Medications Among Older Adults Worldwide: A Systematic Review and Meta-Analysis
Source: JAMA Netw Open. 2023 Aug 2;6(8):e2326910. doi: 10.1001/jamanetworkopen.2023.26910 (PMC10398411; doi:10.1001/jamanetworkopen.2023.26910)
Supplement: Supplement 1. — eAppendix 1. A Sample of the Search Strategy eAppendix 2. Characteristics of Included Studies eAppendix 3. Risk of Bias Assessment eAppendix 4. Meta-Analysis of the Prevalence of PIMs in Outpatients Ordered by Countries eAppendix 5. Forest Plot of the Overall Prevalence of PIM in Outpatient Services eAppendix 6. Stratified Meta-Analysis of the Trend of the Prevalence of PIM for Each Group in World Bank Countries eAppendix 7. The List of the Top 10 Most Common Drugs Used With PIM Criteria eAppendix 8. Leave-One-Out Sensitivity Analyses eAppendix 9. Funnel Plot for the Prevalence of PIMs eReferences [file jamanetwopen-e2326910-s001.pdf]

Supplementary Online Content

Tian F, Chen Z, Zeng Y, Feng Q, Chen X. Prevalence of use of potentially inappropriate medications among older adults worldwide: a systematic review and meta-analysis. *JAMA Netw Open*. 2023;6(8):e2326910. doi:10.1001/jamanetworkopen.2023.26910

eAppendix 1. A Sample of the Search Strategy
eAppendix 2. Characteristics of Included Studies
eAppendix 3. Risk of Bias Assessment
eAppendix 4. Meta-Analysis of the Prevalence of PIMs in Outpatients Ordered by Countries
eAppendix 5. Forest Plot of the Overall Prevalence of PIM in Outpatient Services
eAppendix 6. Stratified Meta-Analysis of the Trend of the Prevalence of PIM for Each Group in World Bank Countries
eAppendix 7. The List of the Top 10 Most Common Drugs Used With PIM Criteria
eAppendix 8. Leave-One-Out Sensitivity Analyses
eAppendix 9. Funnel Plot for the Prevalence of PIMs
eReferences

This supplementary material has been provided by the authors to give readers additional information about their work.

© 2023 Tian F et al. *JAMA Network Open.*

eAppendix 1. A sample of the search strategy

PubMed

1	"Ambulatory Care"[MeSH]
2	"Outpatient Care"[title/abstract]
3	"Outpatient Health Service*"[title/abstract]
4	"Outpatient Service*"[title/abstract]
5	"Clinic Visit*"[title/abstract]
6	“Urgent Care*”[title/abstract]
7	"Outpatient*"[title/abstract]
8	1 or 2 or 3 or 4 or 5 or 6 or 7
9	"Aged"[MeSH]
10	“elder*”[title/abstract]
11	“Older“[title/abstract]
12	“Geriatric”[title/abstract]
13	9 or 10 or 11 or 12
14	“Potentially Inappropriate Medication”[MeSH]
15	“Inappropriate Prescribing”[MeSH]
16	“Potentially Inappropriate Medication*“[Title/Abstract]
17	“Potentially Inappropriate Prescription*“[Title/Abstract]
18	“Inappropriate Medication“[Title/Abstract]
19	“Inappropriate Prescription”[Title/Abstract]
20	“Inappropriate Prescribing”[Title/Abstract]
21	“Inappropriate Drug Use”[Title/Abstract]
22	“Over Prescribing*”[title/abstract]
23	14 or 15 or 16 or 17 or 18 or 19 or 20 or 21 or 22
24	8 and 13 and 23

Embase

1	exp outpatient
2	outpatient.ab.ti
3	1 or 2
4	exp potentially inappropriate medication

© 2023 Tian F et al. *JAMA Network Open.*

5	potentially inappropriate medication.ab.ti
6	4 or 5
7	exp aged
8	older.ab.ti
9	7 or 8
10	3 and 6 and 9

Web of Science

1	TI=outpatient
2	TI=potentially inappropriate medication
3	TI=older
4	1 and 2 and 3

© 2023 Tian F et al. *JAMA Network Open.*

eAppendix 2. Characteristic of included studies

Study	Country	Location	Study design	Sample period	Age definition of older	Mean age (y)	Sample size	Mean of drugs	Male (%)	Population	PIM criteria applied	Number of patients with 1 + PIM	PIM prevalence	Most commonly used PIM
Asia														
Chang et al. 2018	China	Taiwan	Cross-sectional study	2007.8-2007.10	65	76.2 ± 6.2	193	8.9 ± 3.1	53.4	Outpatients	PIM-Taiwan criteria 2010, PIM-Taiwan criteria 2018	83/131	83/193; 131/193	Benzodiazepines
Chen et al. 2021	China	Hangzhou	Cross-sectional study	2016.1-2016.3	65	73.29 ± 6.53	13221	3.2 ± 2.52	51.3	Outpatients	STOPP/START V2	1040	1040/13221	Benzodiazepines
Huang et al. 2020	China	Changsha	Cross-sectional study	2018.1-2018.12	65	86	1874	4	80	Outpatients	Beers criteria 2019, Chinese criteria 2017	656/949	656/1874; 949/1874	Benzodiazepines
Lin et al. 2011	China	Taiwan	Cross-sectional study	2008.8	65	74.8 ± 5.3	327	NR	49.5	Outpatients	Beers criteria 2003	90	90/327	Orphenadrine
Lu et al. 2022	China	Shenzhen	Cross-sectional study	2015-2017	65	74.8 ± 5.4	74916	NR	55.2	Diabetes outpatients	Beers criteria 2019	27017	27017/74916	Diuretics
Tian et al. 2021	China	Chengdu	Cross-sectional study	2018.1-2018.12	65	74	12005	3	59.61	Outpatients	Beers criteria 2019, Beers criteria 2015	4129/3719	4129/12005; 3719/12005	Benzodiazepines
Tian et al. 2022a	China	Chengdu	Cross-sectional study	2018.1-2018.12	65	72	6160	3	53.47	Cancer outpatients	Chinese criteria 2017, Beers criteria 2019, STOPP/START V2	2117/2011/983	2117/6160; 2011/6160; 983/6160	Benzodiazepines
Tian et al. 2022b	China	Chengdu	Cross-sectional study	2016.1-2021.12	65	NR	7238	NR	NR	Lung cancer outpatients	Beers criteria 2019	2225	2225/7238	NR
Weng et al. 2013	China	Taiwan	Cross-sectional study	2012.1-2012.6	65	75.5 ± 7.1	780	6.7 ± 4.6	43	Chronic diseases outpatients	STOPP/START V1	302	302/780	NR
Zeng et al. 2020	China	Beijing	Cross-sectional study	2018.9-2019.11	65	NR	1519	NR	47.1	Outpatients	Beers criteria 2015, Beers criteria 2019, STOPP/START V2	604/685/911	604/1519; 685/1519; 911/1519	Diuretic
Zhang et al. 2021	China	Hongkon g	Cross-sectional study	2014.1-2014.12	65	75.1 ± 7.7	489301	9.6 ± 7.0	45.6	Outpatients	Beers criteria 2015, Hong Kong-specific criteria	232445/241982	232445/4893 01; 241982/4893 01	Chlorpheniramine
Zhang et al. 2022	China	Chengdu	Cross-sectional study	2016.1-2018.12	60	NR	44458	NR	61.54	Outpatients	Chinese criteria 2017, Beers criteria 2015	15728/13358	15728/44458; 13358/44458	Benzodiazepines
Zhao et al. 2022	China	National	Cross-sectional study	2020.1-2020.12	65	80.88 ± 7.69	18624	2	45.14	Dementia outpatients	Beers criteria 2019	7344	7344/18624	Antipsychotic drugs
Anand et al. 2022	India	New Delhi	Cross-sectional study	2016.4-2017.11	60	65.4 ± 4.7	380	6.7 ± 2.1	56.6	Outpatients	Beers criteria 2015	249	249/380	Proton pump inhibitors
Bahat et al. 2019	India	Kerala	Cross-sectional study	2016.1-2016.6	65	73.6 ± 6.7	400	NR	47.7	Outpatients	Beers criteria 2015	136	136/400	Proton-pump inhibitors
Mugada et al. 2021	India	Visakhapa tnam	Cross-sectional study	2019.1-2020.1	65	65.90 ± 5.48	275	NR	53.1	Outpatients	STOPP/START V2	60	60/275	Diclofenac
Shah et al. 2016	India	Ahmedab ad	Cross-sectional study	2015.2-2015.7	65	68.9 ± 7.1	236	6.11 ± 4.3	NR	Cardiac outpatients	Beers criteria 2012	69	69/236	Spironolactone
Sharma et al. 2021	India	Moga	Cross-sectional study	2018	65	NR	456	3	59.6	Psychiatric outpatients	Beers criteria 2019, STOPP/START V2	362/282	362/456; 282/456	Benzodiazepines
Zaveri et al. 2010	India	Ahmedab ad	Cross-sectional study	2005.11-2006.2	65	NR	407	4.27	53.07	Outpatients	Beers criteria 2003	96	96/407	Pheniramine
Patel et al. 2022	India	Gujarat	Cross-sectional study	2018.10-2019.11	65	69.43 ± 5.47	306	5.04 ± 2.44	60.1	Outpatients	STOPP/START V2	88	88/306	Antihistaminic
Arai et al. 2019	Japan	Chiba	Cross-sectional study	2016.10-2016.12	65	73	13630	3	50.3	Outpatients	STOPP-J criteria	5245	5245/13630	Non-steroidal anti-inflammatory drugs
Bonfiglio et al. 2019	Japan	Nagoya	Cross-sectional study	2019	64	78.3 ± 5.8	160	5.4 ± 4.1	54.4	Dementia outpatients	STOPP-J criteria	54	54/160	Antithrombotic drugs
Fujie et al. 2020	Japan	Ibaraki	Cross-sectional study	2015.2-2015.3	75	NR	8080	4	45.4	Outpatients	STOPP-J criteria	2157	2157/8080	Benzodiazepines

© 2023 Tian F et al. *JAMA Network Open.*

Masumoto et al. 2017	Japan	Tokyo	Cross-sectional study	2016.1-2016.3	65	75.7 ± 7.5	740	4	48.8	Outpatients	STOPP/START V2	239	239/740	Benzodiazepines
Suzuki et al. 2021	Japan	National	Cross-sectional study	2014.10, 2019.12	65	NR	180673; 333869	4.05 ± 3.24; 3.98 ± 3.16	43.9; 43.6	Outpatients	STOPP-J criteria	48420/145900	48420/180673; 145900/333869	NR
Uragami et al. 2021	Japan	Kagawa	Cross-sectional study	2020.2-2020.4	65	70	923	4	46.8	Frailty outpatients	STOPP-J criteria	420	420/923	Benzodiazepines
Bahat et al. 2017	Turkey	Istanbul	Cross-sectional study	2000.6-2014.6	65	77.6 ± 6.3	667	6.1 ± 3.4	36.9	Outpatients	Beers criteria 2012, STOPP/START V2	222/261	222/667; 261/667	Aspirin
Kelleciet et al. 2022	Turkey	Ankara	Cross-sectional study	2015.9-2016.5	65	75.75 ± 6.56	700	7.46 ± 2.38	37.2	Outpatients	STOPP/START V2	316	316/700	Non-steroidal anti-inflammatory drugs
Paksoy et al. 2019	Turkey	Istanbul	Cross-sectional study	2014.12-2015.3	65	71.78 ± 5.50	114	NR	55.26	Oncology outpatients	STOPP/START V2	18	18/114	Aspirin
Pala et al. 2022	Turkey	Istanbul	Cross-sectional study	2018.1-2020.1	65	73.33 ± 7.30	657	5.49 ± 3.93	38.8	Outpatients	STOPP/START V2	424	424/657	Non-steroidal anti-inflammatory drugs
Sahin et al. 2021	Turkey	Ankara	Cross-sectional study	2019.6-2019.12	65	73.95 ± 7.30	235	5.14 ± 1.90	65.1	Psychiatry outpatients	Beers criteria 2019	84	84/235	Antipsychotics
Sari et al. 2021	Turkey	Eskişehir	Cross-sectional study	2017.12-2018.1	65	73.18 ± 5.99	513	5.67 ± 2.51	54.2	Cardiac outpatients	Beers criteria 2019, STOPP/START V2	198/234	198/513; 234/513	Aspirin
Cho et al. 2019	Korea	National	Cross-sectional study	2017.9-2017.12	65	74.2 ± 6.26	2143	6.48 ± 2.46	76.2	Lower urinary tract symptoms outpatients	Beers criteria 2015	1579	1579/2143	NR
Kim et al. 2015	Korea	National	Cross-sectional study	2006.1-2006.6	65	NR	40995267	4.2 ± 3.3	35.5	Outpatients	Beers criteria 2003, Zhan criteria, Canadian criteria	14976293/7922080/4590023	14976293/40995267; 7922080/40995267; 4590023/40995267	NR
Kim et al. 2021	Korea	National	Cross-sectional study	2016	65	73.70 ± 6.56	27062307	NR	40.19	Cardiovascular outpatients	STOPP/START V2	341664	341664/27062307	Non-steroidal anti-inflammatory drugs
Lim et al. 2016	Korea	Seoul	Cross-sectional study	2014.1-2014.12	65	72.4 ± 6.1	25810	NR	44.8	Outpatients	Beers criteria 2012	7132	7132/25810	Benzodiazepines
Yoon et al. 2022	Korea	Seoul	Cross-sectional study	2016.1-2016.12	65	77	2100	6	36.9	Dementia outpatients	Beers criteria 2015	987	987/2100	Benzodiazepines
Al-Omar et al. 2013	Saudi	Riyadh	Cross-sectional study	2002-2004	65	74.02 ± 7.46	910644	NR	58.92	Outpatients	Beers criteria 2003	20521	20521/910644	Digoxin
Alwhaibi et al. 2022	Saudi	Riyadh	Cross-sectional study	2019.1-2020.1	65	72 ± 6.16	1853	7	38.32	Diabetes and hypertension outpatients	Beers criteria 2019	1039	1039/1853	Gastrointestinal medications
Alhawassi et al. 2019	Saudi	Riyadh	Cross-sectional study	2016.1-2016.12	65	72.6 ± 6.2	4073	NR	43.2	Outpatients	Beers criteria 2015	2309	2309/4073	Gastrointestinal medications
Meraya et al. 2020	Saudi	Jazan	Cross-sectional study	2018.1-2018.12	60	NR	1300	NR	41.4	Psychiatric disorders outpatients	Beers criteria 2015	892	892/1300	Amitriptyline
Europe														
Cojutti et al. 2016	Italy	Friuli-Venezia Giulia	Cross-sectional study	2014.3	65	76	527	5	42.7	Outpatients	Beers criteria 2012	292	292/527	Benzodiazepines
Galimberti et al. 2022	Italy	Piedmont	Cross-sectional study	2012-2018	65	75.69 ± 7.17; 75.77 ± 7.25; 75.83 ± 7.29; 75.89 ± 7.29; 75.98 ± 7.32; 76.05 ± 7.32;	967268/984352/1001662/1007880/1016257/1019764/102215	6	38.61,38.94,38.77,38.84,38.85,38.98,39.03	Outpatients	ERD list	418537/429670/408378/389848/375812/363138/339764	418537/967268; 429670/984352;	Diclofenac

© 2023 Tian F et al. *JAMA Network Open.*

						76.14 ± 7.34	4						408378/1001662; 389848/1007880; 375812/1016257; 363138/1019764; 3397641022154	
Loddo et al. 2022	Italy	Monserrato	Cross-sectional study	2018.1-2018.12	65	80	3091	7	28.3	Outpatients	Beers criteria 2019, STOPP/START V2	2287/2358	2287/3091; 2358/3091	NR
Maio et al. 2006	Italy	Emilia Romagna	Cohort study	2001.1-2001.12	65	75.6 ± 7.5	849425	NR	41.1	Outpatients	Beers criteria 2003	152641	152641/849425	Doxazosin
Maio et al. 2010	Italy	Parma	Cohort study	2006.1-2006.12	65	75.7 ± 7.7	91741	NR	40.9	Outpatients	Beers criteria 2003	23662	23662/91741	Non-steroidal anti-inflammatory drugs
Varga et al. 2017	Italy	Emilia Romagna	Cohort study	2003.1-2013.12	65	NR	1480137	NR	NR	Outpatients	Maio criteria	802815	802815/1480137	Non-steroidal anti-inflammatory drugs
Bongue et al. 2009	France	Nancy-Vandoeuvre, Verdun, Longwy	Cross-sectional study	1995-2004	65	70.1 ± 4.3	30683	3.3 ± 2.6	48.8	Outpatients	Beers criteria 1997, French criteria	3097/7802	3097/30683; 7802/30683	Propoxyphene
Choukroun et al. 2020	France	Nimes	Cross-sectional study	2016.5-2017.3	75	83	51	NR	43	Cancer outpatients	French criteria, STOPP/START V2	16/29	16/83; 29/83	NR
Drusch et al. 2021	France	National	Cross-sectional study	2011, 2013,2015, 2017, 2019	75	82.1 ± 5.4; 82.4 ± 5.5; 82.6 ± 5.6; 82.8 ± 5.7; 82.8 ± 5.8	5777645/6068742/6151861/6206973/6328155	NR	37.8, 38.5, 38.9, 39.5, 40.1	Outpatients	Beers criteria 2015, STOPP/START V2	2884533/2872892/2776084/2639705/2506266	2884533/5777645; 2872892/6068742; 2776084/6151861; 2639705/6206973; 2506266/6328155	Benzodiazepines
Dubois-Puechlong et al. 2019	France	Multicenter	Cross-sectional study	2016.3-2016.5	75	83 ± 5.5	1178	7	41	Antithrombotic therapies outpatients	STOPP/START V2	235	235/1178	NR
Dormann et al. 2013	Germany	Bavaria	Cross-sectional study	2010.9	65	78±7	351	NR	43.9	Outpatients	PRISCUS list	51	51/351	NR
Endres et al. 2016	Germany	NR	Cross-sectional study	2019.1-2020.12	65	73	392337	4.3 ± 3.5	39.3	Outpatients	PRISCUS list	79041	79041/392337	Benzodiazepines
Fiss et al. 2011	Germany	Mecklenburg	Cross-sectional study	2006.3-2008.12	65	80.5 ± 6.7	744	8.2 ± 4	26.9	Outpatients	Beers criteria 2003	134	134/744	Benzodiazepines
Gudd et al. 2020	Germany	Bavaria	Cross-sectional study	2010-2014	65	NR	410934	NR	NR	Outpatients	PRISCUS list	23021	23021/410934	Hypnotic
Ham et al. 2022	Netherlands	National	Cohort study	2009-2014	65	74	7864	6	67	Lung cancer outpatients	OncPal	3538	3538/7864	Peptic ulcer prophylaxis
Boersma et al. 2019	Netherlands	Utrecht	Cluster-randomized controlled trial	2014.10-2016.7	70	77.8 ± 5.7/79.0 ± 6.0	124	9	51.6	Preoperative outpatients	STOPP/START V2	118	118/124	Proton-pump inhibitors
Oktora et al. 2021	Netherlands	Groningen	Cross-sectional study	2012-2016	65	NR	16458/17077/17439/17670/15267	NR	NR	Diabetes outpatients	Beers criteria 2015	3004/3057/3098/3053/2518	3004/16458; 3057/17077; 3098/17439; 3053/17670;	Proton-pump inhibitors

© 2023 Tian F et al. *JAMA Network Open.*

													2518/15267	
van et al. 2021	Netherlands	Deventer	Cross-sectional study	2018.5-2019.1	65	72 ± 8	150	11	59	Cancer outpatients	STOPP/START V2	97	97/150	Benzodiazepines
Santos-Pérez et al. 2021	Spain	Soria, Valladolid	Cross-sectional study	2016.3	65	78.0 ± 7.2	225	NR	48	Psychotropic outpatients	Beers criteria 2015	51	51/225	Benzodiazepines
Yeste-Gómez et al. 2014	Spain	Madrid	Cross-sectional study	2012.10-2012.12	65	80.2	131	NR	58.8	Outpatients	STOPP/START V1	73	73/131	Benzodiazepines
Cvetković et al. 2019	Serbia	Belgrade	Cross-sectional study	2019	65	74.23 ± 6.92	248	5.25 ± 2.70	62.1	Outpatients	STOPP/START V2	53	53/248	Proton-pump inhibitors
Projovic et al. 2016	Serbia	Kragujevac	Case-control study	2013.9-2014.9	65	NR	324	NR	38.9	Outpatients	STOPP/START V2	122	122/324	Benzodiazepines
North America														
Buck et al. 2009	USA	Utah, Cleveland	Cross-sectional study	2006	65	NR	61251	NR	40.1	Outpatients	Beers criteria 2003; Zhan Criteria	14221/10196	14221/61251; 10196/61251	Fluoxetine
Curtis et al. 2004	USA	National	Cohort study	1999	65	73.7 ± 6.5	765423	NR	41.7	Outpatients	Beers criteria 1997	162370	162370/765423	Amitriptyline
Dunn et al. 2011	USA	Oklahoma	Cross-sectional study	2008.11-2009.3	65	74	120	NR	NR	Outpatients	Beers criteria 2003	45	45/120	Estrogen formulations
Gardner et al. 2020	USA	Colorado	Cross-sectional study	2017.3-2017.8	65	NR	192	NR	40.6	Outpatients	Beers criteria 2019	58	58/192	NR
Hustey et al. 2007	USA	Cleveland	Cross-sectional study	2004.6	65	75.13 ±7.05	352	8.5	45	Outpatients	Beers criteria 2003	111	111/352	Propoxyphene/acetaminophen
Lund et al. 2011	USA	Iowa	Cross-sectional study	2001-2003	65	74.6 ± 5.4	240	10.5 ± 4.0	98.3	Outpatients	Beers criteria 2003	106	106/240	NR
Maggiore et al. 2014	USA	Multicenter	Cohort study	2006.11-2009.11	65	73	500	5 ± 4	44	Cancer outpatients	Beers criteria 2012; Zhan Criteria	147/54	147/500; 54/500	Zolpidem
Maio et al. 2006	USA	Pennsylvania	Cross-sectional study	2004.1-2004.6	65	75.0 ± 6.7	100	NR	18	Outpatients	Beers criteria 2003	24	24/100	Non-steroidal anti-inflammatory drugs
Nightingale et al. 2022	USA	Pennsylvania	Cross-sectional study	2017.11-2020.2	60	67.9 ± 6.4	168	NR	24.4	Diabetes outpatients	Beers criteria 2019	67	67/168	Non-steroidal anti-inflammatory drugs
Pugh et al. 2005	USA	National	Cross-sectional study	1999.10-2000.10	65	73.5 ± 5.6	1265434	NR	98	Outpatients	Zhan Criteria	249566	249566/1265434	Chlordiazepoxide
Pyszka et al. 2010	USA	Wisconsin	Cross-sectional study	2008.4-2008.9	70	78.4 ± 5.4	111	NR	99.1	Outpatients	STOPP/START V1	65	65/111	Aspirin
Skaar et al. 2017	USA	National	Cross-sectional study	2012	65	NR	17663006	NR	NR	Dental outpatients	Beers criteria 2015	10445148	10445148/17663006	Omeprazole
Viswanathan et al. 2005	USA	National	Cross-sectional study	2001	65	NR	157680651	NR	40.8	Outpatients	Beers criteria 2012	21168138	21168138/157680651	Propoxyphene
Weston et al. 2010	USA	California	Cross-sectional study	2008.7-2009.1	NR	75.3 ± 9.8	689	NR	50.5	Mild cognitive impairment outpatients	Beers criteria 2003	143	143/689	Anticholinergics
South America														
Baldoni et al. 2014	Brazil	Sao Paulo	Cross-sectional study	2008.11-2009.5	60	69.8 ± 6.5	1000	6.9	33.9	Outpatients	Beers criteria 2003; Beers criteria 2012	480/592	480/1000; 592/1000	Diclofenac
da Costa et al. 2019	Brazil	Fortaleza	Cross-sectional study	2017.4-2017.9	60	66.5 ± 5.3	143	NR	65.7	Kidney transplant outpatients	Beers criteria 2015	111	111/143	Omeprazole
Faustino et al. 2013	Brazil	Sao Paulo	Cross-sectional study	2008.2-2008.5	60	80.1	1270	NR	23	Outpatients	Beers criteria 2003	342	342/1270	Muscle relaxant
Fulone et al. 2017	Brazil	Sao Paulo	Cross-sectional study	2008.1-2009.12	60	NR	174	NR	18.9	Taking antidepressant outpatients	Beers criteria 2015; STOPP/START V1	52/69	52/174; 69/174	Benzodiazepines
Marques et al.	Brazil	Mato	Cross-sectional	2016.3-2017.9	60	69.5 ± 6.79	44	NR	36.4	Outpatients	Beers criteria 2015	10	10/44	Insulin

© 2023 Tian F et al. *JAMA Network Open.*

2018		Grosso do Sul	study											
Oliveira et al. 2020	Brazil	Sao Paulo	Cross-sectional study	2020	60	74.9 ± 9.4	233	6.6 ± 3.1	NR	Outpatients	PIM-CCVAEs list	173	173/233	NR
Reis et al. 2017	Brazil	Minas Gerais	Cross-sectional study	2015.5-2015.12	60	NR	160	3	42.5	Cancer outpatients	Beers criteria 2015	78	78/160	Proton-pump inhibitors
Viana et al. 2022	Brazil	Sao Paulo	Cohort study	2014-2017	60	79.2±8.2	868	8.5	38.8	Acute conditions outpatients	Beers criteria 2019	352	352/868	Proton-pump inhibitors
Africa														
abubakar et al. 2021	Nigeria	Niger	Cross-sectional study	2016.6-2016.9	65	71.1 ± 6.1	244	5.4 ± 2.3	49.2	Outpatients	Beers criteria 2015; STOPP/START V2	164/98	164/244; 98/244	Non-steroidal anti-inflammatory drugs
Akande-Sholabi et al. 2018	Nigeria	Ibadan	Cross-sectional study	2016.6-2016.9	60	70.2 ± 5.9	400	NR	40	Outpatients	Beers criteria 2015	124	124/400	Non-steroidal anti-inflammatory drugs
Fadare et al. 2013	Nigeria	Ido-Ekiti	Cross-sectional study	2011.4-2011.6	65	72.8 ± 7.2	220	3.8 ± 1.3	41.8	Outpatients	Beers criteria 2012	56	56/220	Non-steroidal anti-inflammatory drugs
Saka et al. 2019	Nigeria	NR	Cross-sectional study	2016.1-2016.12	60	69.03 ± 7.35	352	4.60 ± 1.65	42.3	Outpatients	Beers criteria 2015	124	124/352	Antidepressants
Bhagavathula et al. 2021	Ethiopia	NR	Cross-sectional study	2020.3-2020.8	65	71.9 ± 6.07	320	3.4 ± 1.69	59	Outpatients	Beers criteria 2019	151	151/320	Antidiabetic medications
Lemma et al. 2020	Ethiopia	Addis Ababa	Cross-sectional study	2019.7-2019.8	60	NR	400	2.51	55	Outpatients	Beers criteria 2019	188	188/400	Analgesics
Tesfaye et al. 2020	Ethiopia	Jimma	Cross-sectional study	2020.11-2020.12	65	70	219	4	65.3	Outpatients	Beers criteria 2019 STOPP/START V2	182/99	182/219; 99/219	Aspirin/Amitriptyline
Oceania														
Cross et al. 2016	Australia	Multicenter	Cross-sectional study	2005.4-2007.8	NR	77.6 ± 7.4	964	NR	52.7	Memory outpatients	PIMcog	206	206/964	Anticholinergics
Saarelainen et al. 2014	Australia	Adelaide	Cross-sectional study	2009.1-2010.7	70	76.7 ± 4.8	385	NR	59	Cancer outpatients	Beers criteria 2012	102	102/385	Benzodiazepines

Note: NR: not reported

© 2023 Tian F et al. *JAMA Network Open.*

eAppendix 3. Risk of bias assessment

Study	1.Target population a close representation of the national population?	2. Sampling frame representative?	3. Random selection used?	4. Data collected directly from subjects?	5. Acceptable case definition used?	6. Same mode of data collection for all?	7. Were the numerator(s) and denominator(s) for the parameter of interest appropriate?
Chang et al. 2018	N	Y	NA	Y	Y	Y	Y
Chen et al. 2021	N	N	NA	Y	Y	Y	Y
Huang et al. 2020	N	Y	NA	Y	Y	Y	Y
Lin et al. 2011	N	Y	NA	Y	Y	Y	Y
Lu et al. 2022	N	N	NA	Y	Y	Y	Y
Tian et al. 2021	N	Y	Y	Y	Y	Y	Y
Tian et al. 2022a	N	N	Y	Y	Y	Y	Y
Tian et al. 2022b	N	N	Y	Y	Y	Y	N
Weng et al. 2013	N	N	NA	Y	Y	Y	Y
Zeng et al. 2020	N	Y	NA	Y	Y	Y	Y
Zhang et al. 2021	Y	Y	NA	Y	Y	Y	Y
Zhang et al. 2022	N	Y	Y	Y	Y	Y	Y
Zhao et al. 2022	Y	N	Y	Y	Y	Y	N
Anand et al. 2022	N	Y	NA	Y	Y	Y	Y
Bahat et al. 2019	N	Y	Y	Y	Y	Y	Y
Mugada et al. 2021	N	Y	Y	Y	Y	Y	Y
Shah et al. 2016	N	N	NA	Y	Y	Y	Y
Sharma et al. 2021	N	N	NA	Y	Y	Y	N
Zaveri et al. 2010	N	Y	NA	Y	Y	Y	Y
Patel et al. 2022	N	Y	NA	Y	Y	Y	Y
Arai et al. 2019	N	Y	NA	Y	Y	Y	N
Bonfiglio et al. 2019	N	N	NA	Y	Y	Y	Y
Fujie et al. 2020	N	Y	NA	Y	Y	Y	Y
Masumoto et al. 2017	N	Y	NA	Y	Y	Y	Y
Suzuki et al. 2021	Y	Y	NA	Y	Y	Y	Y
Uragami et al. 2021	N	N	Y	Y	Y	Y	Y
Bahat et al. 2017	N	Y	Y	Y	Y	Y	Y
Kelleciet al. 2022	N	Y	NA	Y	Y	Y	Y
Paksoy et al. 2019	N	N	NA	Y	Y	Y	Y
Pala et al. 2022	N	Y	NA	Y	Y	Y	Y
Sahin et al. 2021	N	N	NA	Y	Y	Y	Y
Sari et al. 2021	N	N	NA	Y	Y	Y	Y
Cho et al. 2019	Y	N	NA	Y	Y	Y	Y
Kim et al. 2015	Y	Y	NA	Y	Y	Y	Y
Kim et al. 2021	Y	N	Y	Y	Y	Y	Y
Lim et al. 2016	N	N	NA	Y	Y	Y	Y
Yoon et al. 2022	N	N	NA	Y	Y	Y	Y
Al-Omar et al. 2013	Y	Y	NA	Y	Y	Y	Y

© 2023 Tian F et al. *JAMA Network Open.*

Alwhaibi et al. 2022	N	N	NA	Y	Y	Y	Y
Alhawassi et al. 2019	N	Y	NA	Y	Y	Y	Y
Meraya et al. 2020	N	N	NA	Y	Y	Y	Y
Cojutti et al. 2016	Y	Y	NA	Y	Y	Y	Y
Galimberti et al. 2022	N	Y	NA	Y	Y	Y	Y
Loddo et al. 2022	N	Y	NA	Y	Y	Y	N
Maio et al. 2006	Y	Y	NA	Y	Y	Y	Y
Maio et al. 2010	N	Y	NA	Y	Y	Y	Y
Varga et al. 2017	N	Y	NA	Y	Y	Y	Y
Bongue et al. 2009	Y	Y	NA	Y	Y	Y	Y
Choukroun et al. 2020	N	N	NA	Y	Y	Y	Y
Drusch et al. 2021	Y	Y	NA	Y	Y	Y	Y
Dubois-Puechlong et al. 2019	Y	N	NA	Y	Y	Y	Y
Dormann et al. 2013	N	N	NA	Y	Y	Y	Y
Endres et al. 2016	N	Y	NA	Y	Y	Y	Y
Fiss et al. 2011	N	Y	NA	Y	Y	Y	Y
Gudd et al. 2020	N	Y	NA	Y	Y	Y	Y
Ham et al. 2022	Y	N	NA	Y	Y	Y	N
Boersma et al. 2019	N	N	Y	Y	Y	Y	Y
Oktora et al. 2021	N	N	NA	Y	Y	Y	Y
van et al. 2021	N	N	NA	Y	Y	Y	Y
Santos-Pérez et al. 2021	N	N	Y	Y	Y	Y	Y
Yeste-Gómez et al. 2014	N	Y	NA	Y	Y	Y	Y
Cvetković et al. 2019	N	Y	NA	Y	Y	Y	Y
Projovic et al. 2016	N	Y	NA	Y	Y	Y	Y
Buck et al. 2009	N	Y	NA	Y	Y	Y	Y
Curtis et al. 2004	Y	Y	NA	Y	Y	Y	Y
Dunn et al. 2011	N	Y	NA	Y	Y	Y	Y
Gardner et al. 2020	N	Y	Y	Y	Y	Y	Y
Hustey et al. 2007	N	Y	NA	Y	Y	Y	Y
Lund et al. 2011	N	Y	Y	Y	Y	Y	N
Maggiore et al. 2014	N	N	Y	Y	Y	Y	Y
Maio et al. 2006	N	Y	Y	Y	Y	Y	Y
Nightingale et al. 2022	N	N	NA	Y	Y	Y	Y
Pugh et al. 2005	Y	Y	NA	Y	Y	Y	Y
Pyszka et al. 2010	N	Y	NA	Y	Y	Y	Y
Skaar et al. 2017	Y	N	NA	Y	Y	Y	N
Viswanathan al. 2005	Y	Y	NA	Y	Y	Y	Y
Weston et al. 2010	N	N	NA	Y	Y	Y	Y
Baldoni et al. 2014	N	Y	NA	Y	Y	Y	Y
da Costa et al. 2019	N	N	NA	Y	Y	Y	Y
Faustino et al. 2013	N	Y	NA	Y	Y	Y	Y
Fulone et al. 2017	N	N	NA	Y	Y	Y	Y
Marques et al. 2018	N	Y	NA	Y	Y	Y	Y

© 2023 Tian F et al. *JAMA Network Open.*

Oliveira et al. 2020	N	Y	NA	Y	Y	Y	Y
Reis et al. 2017	N	N	NA	Y	Y	Y	Y
Viana et al. 2022	N	N	NA	Y	Y	Y	Y
abubakar et al. 2021	N	Y	NA	Y	Y	Y	Y
Akande-Sholabi et al. 2018	N	Y	NA	Y	Y	Y	Y
Fadare et al. 2013	N	Y	NA	Y	Y	Y	Y
Saka et al. 2019	N	Y	Y	Y	Y	Y	Y
Bhagavathula et al. 2021	N	Y	Y	Y	Y	Y	Y
Lemma et al. 2020	N	Y	NA	Y	Y	Y	Y
Tesfaye et al. 2020	N	Y	Y	Y	Y	Y	Y
Cross et al. 2016	N	N	Y	Y	Y	Y	Y
Saarelainen et al. 2014	N	N	NA	Y	Y	Y	Y

© 2023 Tian F et al. *JAMA Network Open.*

eAppendix 4. Meta-analysis of the prevalence of PIMs in outpatients ordered by countries

Country	Number of studies (number of data points)	Pooled prevalence of PIMs	95% CI	I2 (%)	*P*
Asia					
China	13 (22)	0.372	0.315-0.431	99.93	<.001
India	7 (8)	0.428	0.274-0.589	98.82	<.01
Japan	6 (7)	0.352	0.295-0.410	99.96	<.001
Turkey	6 (8)	0.396	0.302-0.494	96.67	<.01
Korea	5 (7)	0.279	0.109-0.491	100	<.001
Saudi	4 (4)	0.236	0.188-0.288	74.86	<.001
Europe					
Italy	6 (13)	0.441	0.347-0.538	100	<.001
France	4 (10)	0.327	0.239-0.421	100	<.001
Germany	4 (4)	0.14	0.077-0.217	99.99	<.001
Netherlands	4 (8)	0.362	0.159-0.594	99.78	<.001
Spain	2 (2)	0.384	0.104-0.715	97.49	<.01
Serbia	2 (2)	0.292	0.148-0.462	94.51	<.01
North America					
USA	14 (16)	0.29	0.221-0.363	100	<.001
South America					
Brazil	8 (10)	0.469	0.351-0.589	98.03	<.01
Africa					
Nigeria	4 (5)	0.396	0.259-0.541	96.44	<.01
Ethiopia	3 (4)	0.563	0.371-0.746	97.3	<.01
Oceania					
Australia	2 (2)	0.236	0.188-0.288	74.86	.05

© 2023 Tian F et al. *JAMA Network Open.*

eAppendix 5. Forest plot of the overall prevalence of PIM in outpatient services

Source	Events	Total	Proportion (95% CI)
Chang et al. 2018−1	83	193	0.430 [0.359; 0.503]
Chang et al. 2018−2	131	193	0.679 [0.608; 0.744]
Chen et al. 2021	1040	13221	0.079 [0.074; 0.083]
Huang et al. 2020−1	656	1874	0.350 [0.328; 0.372]
Huang et al. 2020−2	949	1874	0.506 [0.484; 0.529]
Lin et al. 2011	90	327	0.275 [0.228; 0.327]
Lu et al. 2022	27017	74916	0.361 [0.357; 0.364]
Tian et al. 2021−1	4129	12005	0.344 [0.335; 0.353]
Tian et al. 2021−2	3719	12005	0.310 [0.302; 0.318]
Tian et al. 2022a−1	2117	6160	0.344 [0.332; 0.356]
Tian et al. 2022a−2	2011	6160	0.326 [0.315; 0.338]
Tian et al. 2022a−3	983	6160	0.160 [0.151; 0.169]
Tian et al. 2022b	2225	7238	0.307 [0.297; 0.318]
Wong et al. 2013	302	780	0.387 [0.353; 0.422]
Zeng et al. 2020−1	604	1519	0.398 [0.373; 0.423]
Zeng et al. 2020−2	685	1519	0.451 [0.426; 0.476]
Zeng et al. 2020−3	911	1519	0.600 [0.575; 0.624]
Zhang et al. 2021−1	232445	489301	0.475 [0.474; 0.476]
Zhang et al. 2021−2	241982	489301	0.495 [0.493; 0.496]
Zhang et al. 2022−1	15728	44458	0.354 [0.349; 0.358]
Zhang et al. 2022−2	13358	44458	0.300 [0.296; 0.305]
Zhao et al. 2022	7344	18624	0.394 [0.387; 0.401]
Anand et al. 2022	249	380	0.655 [0.605; 0.703]
Bahat et al. 2019	136	400	0.340 [0.294; 0.389]
Mugada et al. 2021	60	275	0.218 [0.171; 0.272]
Shah et al. 2016	69	236	0.292 [0.235; 0.355]
Sharma et al. 2021−1	362	456	0.794 [0.754; 0.830]
Sharma et al. 2021−2	282	456	0.618 [0.572; 0.663]
Zaveri et al. 2010	96	407	0.236 [0.195; 0.280]
Patel et al. 2022	88	306	0.288 [0.238; 0.342]
Arai et al. 2019	5245	13630	0.385 [0.377; 0.393]
Bonfiglio et al. 2019	54	160	0.338 [0.265; 0.416]
Fujie et al. 2020	2157	8080	0.267 [0.257; 0.277]
Masumoto et al. 2017	239	740	0.323 [0.289; 0.358]
Suzuki et al. 2021−1	48420	180673	0.268 [0.266; 0.270]
Suzuki et al. 2021−2	145900	333869	0.437 [0.435; 0.439]
Uragami et al. 2021	420	923	0.455 [0.423; 0.488]
Bahat et al. 2017−1	222	667	0.333 [0.297; 0.370]
Bahat et al. 2017−2	261	667	0.391 [0.354; 0.430]
Kelleciel et al. 2022	316	700	0.451 [0.414; 0.489]
Paksoy et al. 2019	18	114	0.158 [0.096; 0.238]
Pala et al. 2022	424	657	0.645 [0.607; 0.682]
Sahin et al. 2021	84	235	0.357 [0.296; 0.422]
Sari et al. 2021−1	198	513	0.386 [0.344; 0.430]
Sari et al. 2021−2	234	513	0.456 [0.412; 0.500]
Cho et al. 2019	1579	2143	0.737 [0.718; 0.755]
Kim et al. 2015−1	14976293	40995267	0.365 [0.365; 0.365]
Kim et al. 2015−2	7922080	40995267	0.193 [0.193; 0.193]
Kim et al. 2015−3	4590023	40995267	0.112 [0.112; 0.112]
Kim et al. 2021	341664	27062307	0.013 [0.013; 0.013]
Lim et al. 2016	7132	25810	0.276 [0.271; 0.282]
Yoon et al. 2022	987	2100	0.470 [0.448; 0.492]
Al−Omar et al. 2013	20521	910644	0.023 [0.022; 0.023]
Alwhaibi et al. 2022	1039	1853	0.561 [0.538; 0.583]
Alhawassi et al. 2019	2309	4073	0.567 [0.552; 0.582]
Meraya et al. 2020	892	1300	0.686 [0.660; 0.711]
Cojutti et al. 2016	292	527	0.554 [0.510; 0.597]
Galimberti et al. 2022−1	418537	967268	0.433 [0.432; 0.434]
Galimberti et al. 2022−2	429670	984352	0.437 [0.436; 0.437]
Galimberti et al. 2022−3	408378	1001662	0.408 [0.407; 0.409]
Galimberti et al. 2022−4	389848	1007880	0.387 [0.386; 0.388]
Galimberti et al. 2022−5	375812	1016257	0.370 [0.369; 0.371]
Galimberti et al. 2022−6	363138	1019764	0.356 [0.355; 0.357]
Galimberti et al. 2022−7	339764	1022154	0.332 [0.331; 0.333]
Loddo et al. 2022−1	2287	3091	0.740 [0.724; 0.755]
Loddo et al. 2022−2	2358	3091	0.763 [0.747; 0.778]
Maio et al. 2006	152641	849425	0.180 [0.179; 0.181]
Maio et al. 2010	23662	91741	0.258 [0.255; 0.261]
Varga et al. 2017	802815	1480137	0.542 [0.542; 0.543]
Bongue et al. 2009−1	3097	30683	0.101 [0.098; 0.104]
Bongue et al. 2009−2	7802	30683	0.254 [0.249; 0.259]
Choukroun et al. 2020−1	16	83	0.193 [0.114; 0.294]
Choukroun et al. 2020−2	29	83	0.349 [0.248; 0.462]
Drusch et al. 2021−1	2884533	5777645	0.499 [0.499; 0.500]
Drusch et al. 2021−2	2872892	6068742	0.473 [0.473; 0.474]
Drusch et al. 2021−3	2776084	6151861	0.451 [0.451; 0.452]
Drusch et al. 2021−4	2639705	6206973	0.425 [0.425; 0.426]
Drusch et al. 2021−5	2506266	6328155	0.396 [0.396; 0.396]
Dubois−Puechlong et al. 2019	235	1178	0.199 [0.177; 0.223]
Dormann et al. 2013	51	351	0.145 [0.110; 0.187]
Endres et al. 2016	79041	392337	0.201 [0.200; 0.203]
Fiss et al. 2011	134	744	0.180 [0.153; 0.210]
Gudd et al. 2020	23021	410934	0.056 [0.055; 0.057]
Ham et al. 2022	3538	7864	0.450 [0.439; 0.461]
Boersma et al. 2019	118	124	0.952 [0.898; 0.982]
Oktora et al. 2021−1	3004	16458	0.183 [0.177; 0.189]
Oktora et al. 2021−2	3057	17077	0.179 [0.173; 0.185]
Oktora et al. 2021−3	3098	17439	0.178 [0.172; 0.183]
Oktora et al. 2021−4	3053	17670	0.173 [0.167; 0.178]
Oktora et al. 2021−5	2518	15267	0.165 [0.159; 0.171]
van et al. 2021	97	150	0.647 [0.565; 0.723]
Santos−Pérez et al. 2021	51	225	0.227 [0.174; 0.287]
Yeste−Gómez et al. 2014	73	131	0.557 [0.468; 0.644]
Cvetkovic et al. 2019	53	248	0.214 [0.164; 0.270]
Projovic et al. 2016	122	324	0.377 [0.324; 0.432]
Buck et al. 2009−1	14221	61251	0.232 [0.229; 0.236]
Buck et al. 2009−2	10196	61251	0.166 [0.164; 0.169]
Curtis et al. 2004	162370	765423	0.212 [0.211; 0.213]
Dunn et al. 2011	45	120	0.375 [0.288; 0.468]
Gardner et al. 2020	58	192	0.302 [0.238; 0.372]
Hustey et al. 2007	111	352	0.315 [0.267; 0.367]
Lund et al. 2011	106	240	0.442 [0.378; 0.507]
Maggiore et al. 2014−1	147	500	0.294 [0.254; 0.336]
Maggiore et al. 2014−2	54	500	0.108 [0.082; 0.139]
Maio et al. 2006	24	100	0.240 [0.160; 0.336]
Nightingale et al. 2022	67	168	0.399 [0.324; 0.477]
Pugh et al. 2005	249566	1265434	0.197 [0.197; 0.198]
Pyszka et al. 2010	65	111	0.586 [0.488; 0.678]
Skaar et al. 2017	10445148	17663006	0.591 [0.591; 0.592]
Viswanathan et al. 2005	21168138	157680651	0.134 [0.134; 0.134]
Weston et al. 2010	143	689	0.208 [0.178; 0.240]
Baldoni et al. 2014−1	480	1000	0.480 [0.449; 0.511]
Baldoni et al. 2014−2	592	1000	0.592 [0.561; 0.623]
da Costa et al. 2019	111	143	0.776 [0.699; 0.842]
Faustino et al. 2013	342	1270	0.269 [0.245; 0.295]
Fulone et al. 2017−1	52	174	0.299 [0.232; 0.373]
Fulone et al. 2017−2	69	174	0.397 [0.323; 0.473]
Marques et al. 2018	10	44	0.227 [0.115; 0.378]
Oliveira et al. 2020	173	233	0.742 [0.681; 0.797]
Reis et al. 2017	78	160	0.487 [0.408; 0.568]
Viana et al. 2022	352	868	0.406 [0.373; 0.439]
abubakar et al. 2021−1	164	244	0.672 [0.609; 0.731]
abubakar et al. 2021−2	98	244	0.402 [0.340; 0.466]
Akande−Sholabi et al. 2018	124	400	0.310 [0.265; 0.358]
Fadare et al. 2013	56	220	0.255 [0.198; 0.317]
Saka et al. 2019−1	124	352	0.352 [0.302; 0.405]
Bhagavathula et al. 2021	151	320	0.472 [0.416; 0.528]
Lemma et al. 2020	188	400	0.470 [0.420; 0.520]
Tesfaye et al. 2020−1	182	219	0.831 [0.775; 0.878]
Tesfaye et al. 2020−2	99	219	0.452 [0.385; 0.521]
Cross et al. 2016	206	964	0.214 [0.188; 0.241]
Saarelainen et al. 2014	102	385	0.265 [0.222; 0.312]
Total		371224692	0.367 [0.334; 0.400]

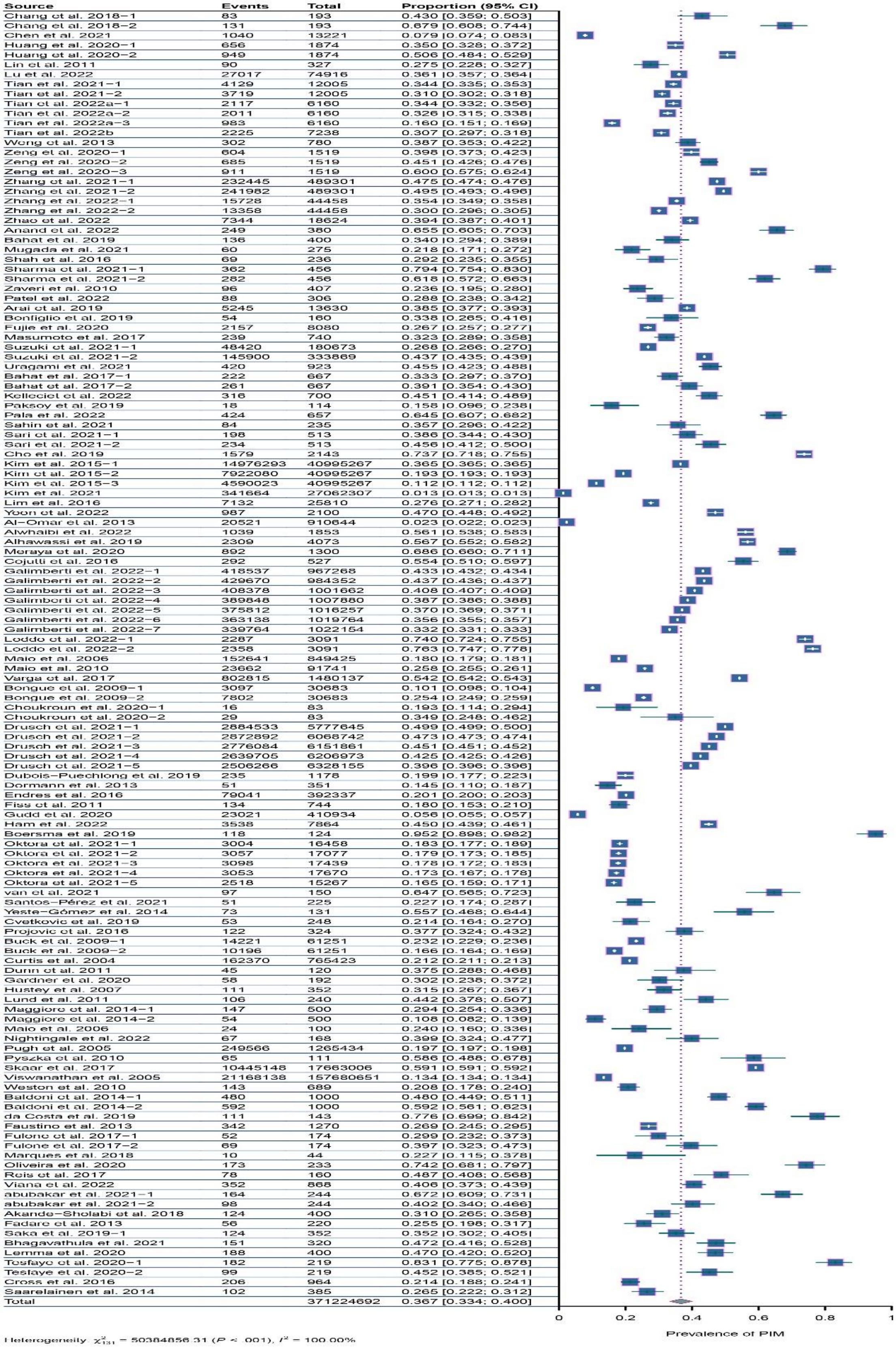

Heterogeneity: $\chi^2_{131} = 50384856.31$ ($P < .001$), $I^2 = 100.00\%$

© 2023 Tian F et al. *JAMA Network Open.*

eAppendix 6. Stratified meta-analysis of the trend of the prevalence of PIM for each group in world bank countries

**Characteristics**	**Pooled prevalence of PIMs**	**95% CI**	**I^2 (%)**	***P***
High				
≤2000	0.187	0.125-0.259	99.92	<.001
2001-2005	0.242	0.141-0.361	100	<.001
2006-2010	0.235	0.168-0.309	100	<.001
2011-2015	0.401	0.304-0.502	100	<.001
2016-2020	0.399	0.321-0.481	100	<.001
Upper-middle				
2006-2010	0.425	0.322-0.532	98.05	<.001
2011-2015	0.426	0.383-0.469	99.88	<.001
2016-2020	0.371	0.290-0.456	99.78	<.001
Lower-middle				
≤2000	0.362	0.306-0.420	78.77	<.001
2011-2015	0.287	0.175-0.414	95.45	<.001
2016-2020	0.464	0.369-0.560	98.08	<.001

eAppendix 7. The list of the top 10 most common drugs used with PIM criteria

Number	Drugs
1	Benzodiazepines
2	Non-steroidal anti-inflammatory drugs
3	Proton pump inhibitors
4	Antidepressants
5	Antipsychotics
6	Anticholinergics
7	Antidiabetic medications
8	Antihistaminic
9	Analgesics
10	Antithrombotic drugs

© 2023 Tian F et al. *JAMA Network Open.*

eAppendix 8. Leave-one-out sensitivity analyses

	proportion	95%-CI	p-value	tau^2	I^2
Omitting Chang et al. 2018-1	0.3662	[0.3331; 0.3999]	0.0405	0.2012	100.00%
Omitting Chang et al. 2018-2	0.3644	[0.3316; 0.3977]	0.0398	0.1994	100.00%
Omitting Chen et al. 2021	0.3694	[0.3367; 0.4027]	0.0395	0.1987	100.00%
Omitting Huang et al. 2020-1	0.3668	[0.3337; 0.4005]	0.0405	0.2013	100.00%
Omitting Huang et al. 2020-2	0.3656	[0.3326; 0.3993]	0.0404	0.2009	100.00%
Omitting Lin et al. 2011	0.3674	[0.3343; 0.4011]	0.0405	0.2011	100.00%
Omitting Lu et al. 2022	0.3667	[0.3336; 0.4005]	0.0405	0.2013	100.00%
Omitting Tian et al. 2021-1	0.3668	[0.3337; 0.4006]	0.0405	0.2013	100.00%
Omitting Tian et al. 2021-2	0.3671	[0.3340; 0.4009]	0.0405	0.2013	100.00%
Omitting Tian et al. 2022a-1	0.3668	[0.3337; 0.4006]	0.0405	0.2013	100.00%
Omitting Tian et al. 2022a-2	0.367	[0.3339; 0.4007]	0.0405	0.2013	100.00%
Omitting Tian et al. 2022a-3	0.3684	[0.3355; 0.4020]	0.0401	0.2002	100.00%
Omitting Tian et al. 2022b	0.3671	[0.3340; 0.4009]	0.0405	0.2013	100.00%
Omitting Weng et al. 2013	0.3665	[0.3334; 0.4002]	0.0405	0.2013	100.00%
Omitting Zeng et al. 2020-1	0.3664	[0.3333; 0.4002]	0.0405	0.2013	100.00%
Omitting Zeng et al. 2020-2	0.366	[0.3330; 0.3997]	0.0405	0.2012	100.00%
Omitting Zeng et al. 2020-3	0.3649	[0.3320; 0.3984]	0.0401	0.2002	100.00%
Omitting Zhang et al. 2021-1	0.3658	[0.3328; 0.3995]	0.0404	0.2011	100.00%
Omitting Zhang et al. 2021-2	0.3657	[0.3327; 0.3994]	0.0404	0.201	100.00%
Omitting Zhang et al. 2022-1	0.3668	[0.3337; 0.4005]	0.0405	0.2013	100.00%
Omitting Zhang et al. 2022-2	0.3672	[0.3341; 0.4009]	0.0405	0.2012	100.00%
Omitting Zhao et al. 2022	0.3665	[0.3334; 0.4002]	0.0405	0.2013	100.00%
Omitting Anand et al. 2022	0.3645	[0.3317; 0.3979]	0.0399	0.1997	100.00%
Omitting Bahat et al. 2019	0.3669	[0.3338; 0.4006]	0.0405	0.2013	100.00%
Omitting Mugada et al. 2021	0.3679	[0.3348; 0.4015]	0.0403	0.2008	100.00%
Omitting Shah et al. 2016	0.3672	[0.3342; 0.4010]	0.0405	0.2012	100.00%
Omitting Sharma et al. 2021-1	0.3633	[0.3310; 0.3964]	0.039	0.1974	100.00%
Omitting Sharma et al. 2021-2	0.3648	[0.3319; 0.3983]	0.04	0.2001	100.00%
Omitting Zaveri et al. 2010	0.3677	[0.3347; 0.4014]	0.0404	0.2009	100.00%
Omitting Patel et al. 2022	0.3673	[0.3342; 0.4010]	0.0405	0.2012	100.00%
Omitting Arai et al. 2019	0.3665	[0.3334; 0.4003]	0.0405	0.2013	100.00%
Omitting Bonfiglio et al. 2019	0.3669	[0.3338; 0.4006]	0.0405	0.2013	100.00%
Omitting Fujie et al. 2020	0.3675	[0.3344; 0.4012]	0.0404	0.2011	100.00%
Omitting Masumoto et al. 2017	0.367	[0.3339; 0.4007]	0.0405	0.2013	100.00%
Omitting Suzuki et al. 2021-1	0.3675	[0.3344; 0.4012]	0.0404	0.2011	100.00%
Omitting Suzuki et al. 2021-2	0.3661	[0.3331; 0.3998]	0.0405	0.2012	100.00%
Omitting Uragami et al. 2021	0.366	[0.3329; 0.3997]	0.0405	0.2012	100.00%
Omitting Bahat et al. 2017-1	0.3669	[0.3338; 0.4007]	0.0405	0.2013	100.00%
Omitting Bahat et al. 2017-2	0.3665	[0.3334; 0.4002]	0.0405	0.2013	100.00%
Omitting Kelleciet et al. 2022	0.366	[0.3330; 0.3997]	0.0405	0.2012	100.00%
Omitting Paksoy et al. 2019	0.3684	[0.3354; 0.4020]	0.0401	0.2003	100.00%
Omitting Pala et al. 2022	0.3646	[0.3318; 0.3980]	0.0399	0.1998	100.00%
Omitting Sahin et al. 2021	0.3667	[0.3336; 0.4005]	0.0405	0.2013	100.00%

© 2023 Tian F et al. *JAMA Network Open.*

Omitting Sari et al. 2021-1	0.3665	[0.3334; 0.4003]	0.0405	0.2013	100.00%
Omitting Sari et al. 2021-2	0.366	[0.3329; 0.3997]	0.0405	0.2012	100.00%
Omitting Cho et al. 2019	0.3638	[0.3312; 0.3970]	0.0394	0.1984	100.00%
Omitting Kim et al. 2015-1	0.3667	[0.3336; 0.4004]	0.0405	0.2013	100.00%
Omitting Kim et al. 2015-2	0.3681	[0.3351; 0.4018]	0.0402	0.2006	100.00%
Omitting Kim et al. 2015-3	0.369	[0.3361; 0.4024]	0.0398	0.1994	100.00%
Omitting Kim et al. 2021	0.3707	[0.3384; 0.4035]	0.0382	0.1955	100.00%
Omitting Lim et al. 2016	0.3674	[0.3343; 0.4011]	0.0405	0.2012	100.00%
Omitting Yoon et al. 2022	0.3659	[0.3328; 0.3996]	0.0404	0.2011	100.00%
Omitting Al-Omar et al. 2013	0.3704	[0.3380; 0.4033]	0.0385	0.1963	100.00%
Omitting Alwhaibi et al. 2022	0.3652	[0.3323; 0.3988]	0.0402	0.2006	100.00%
Omitting Alhawassi et al. 2019	0.3652	[0.3322; 0.3987]	0.0402	0.2005	100.00%
Omitting Meraya et al. 2020	0.3642	[0.3315; 0.3976]	0.0397	0.1992	100.00%
Omitting Cojutti et al. 2016	0.3653	[0.3323; 0.3989]	0.0403	0.2006	100.00%
Omitting Galimberti et al. 2022-1	0.3662	[0.3331; 0.3999]	0.0405	0.2012	100.00%
Omitting Galimberti et al. 2022-2	0.3661	[0.3331; 0.3999]	0.0405	0.2012	100.00%
Omitting Galimberti et al. 2022-3	0.3664	[0.3333; 0.4001]	0.0405	0.2013	100.00%
Omitting Galimberti et al. 2022-4	0.3665	[0.3334; 0.4002]	0.0405	0.2013	100.00%
Omitting Galimberti et al. 2022-5	0.3666	[0.3335; 0.4004]	0.0405	0.2013	100.00%
Omitting Galimberti et al. 2022-6	0.3668	[0.3337; 0.4005]	0.0405	0.2013	100.00%
Omitting Galimberti et al. 2022-7	0.3669	[0.3338; 0.4007]	0.0405	0.2013	100.00%
Omitting Loddo et al. 2022-1	0.3638	[0.3312; 0.3970]	0.0394	0.1984	100.00%
Omitting Loddo et al. 2022-2	0.3636	[0.3311; 0.3967]	0.0392	0.1979	100.00%
Omitting Maio et al. 2006	0.3683	[0.3353; 0.4019]	0.0402	0.2004	100.00%
Omitting Maio et al. 2010	0.3675	[0.3345; 0.4013]	0.0404	0.2011	100.00%
Omitting Varga et al. 2017	0.3653	[0.3324; 0.3990]	0.0403	0.2007	100.00%
Omitting Bongue et al. 2009-1	0.3691	[0.3363; 0.4025]	0.0397	0.1992	100.00%
Omitting Bongue et al. 2009-2	0.3676	[0.3345; 0.4013]	0.0404	0.201	100.00%
Omitting Choukroun et al. 2020-1	0.368	[0.3350; 0.4017]	0.0403	0.2006	100.00%
Omitting Choukroun et al. 2020-2	0.3668	[0.3337; 0.4005]	0.0405	0.2013	100.00%
Omitting Drusch et al. 2021-1	0.3657	[0.3326; 0.3993]	0.0404	0.201	100.00%
Omitting Drusch et al. 2021-2	0.3659	[0.3328; 0.3995]	0.0404	0.2011	100.00%
Omitting Drusch et al. 2021-3	0.366	[0.3330; 0.3997]	0.0405	0.2012	100.00%
Omitting Drusch et al. 2021-4	0.3662	[0.3331; 0.3999]	0.0405	0.2013	100.00%
Omitting Drusch et al. 2021-5	0.3664	[0.3334; 0.4002]	0.0405	0.2013	100.00%
Omitting Dubois-Puechlong et al. 2019	0.3681	[0.3350; 0.4017]	0.0403	0.2006	100.00%
Omitting Dormann et al. 2013	0.3686	[0.3356; 0.4021]	0.04	0.2	100.00%
Omitting Endres et al. 2016	0.368	[0.3350; 0.4017]	0.0403	0.2007	100.00%
Omitting Fiss et al. 2011	0.3682	[0.3352; 0.4019]	0.0402	0.2005	100.00%
Omitting Gudd et al. 2020	0.3697	[0.3371; 0.4029]	0.0392	0.198	100.00%
Omitting Ham et al. 2022	0.366	[0.3330; 0.3997]	0.0405	0.2012	100.00%
Omitting Boersma et al. 2019	0.3617	[0.3301; 0.3938]	0.037	0.1924	100.00%
Omitting Oktora et al. 2021-1	0.3682	[0.3352; 0.4018]	0.0402	0.2005	100.00%
Omitting Oktora et al. 2021-2	0.3683	[0.3353; 0.4019]	0.0402	0.2004	100.00%
Omitting Oktora et al. 2021-3	0.3683	[0.3353; 0.4019]	0.0402	0.2004	100.00%

© 2023 Tian F et al. *JAMA Network Open.*

Omitting Oktora et al. 2021-4	0.3683	[0.3353; 0.4019]	0.0401	0.2004	100.00%
Omitting Oktora et al. 2021-5	0.3684	[0.3354; 0.4020]	0.0401	0.2003	100.00%
Omitting van et al. 2021	0.3646	[0.3318; 0.3981]	0.0399	0.1998	100.00%
Omitting Santos-Pérez et al. 2021	0.3678	[0.3347; 0.4015]	0.0403	0.2009	100.00%
Omitting Yeste-Gómez et al. 2014	0.3653	[0.3323; 0.3989]	0.0403	0.2006	100.00%
Omitting Cvetković et al. 2019	0.3679	[0.3349; 0.4016]	0.0403	0.2008	100.00%
Omitting Projovic et al. 2016	0.3666	[0.3335; 0.4003]	0.0405	0.2013	100.00%
Omitting Buck et al. 2009-1	0.3678	[0.3347; 0.4015]	0.0404	0.2009	100.00%
Omitting Buck et al. 2009-2	0.3684	[0.3354; 0.4020]	0.0401	0.2003	100.00%
Omitting Curtis et al. 2004	0.3679	[0.3349; 0.4016]	0.0403	0.2008	100.00%
Omitting Dunn et al. 2011	0.3666	[0.3335; 0.4003]	0.0405	0.2013	100.00%
Omitting Gardner et al. 2020	0.3672	[0.3341; 0.4009]	0.0405	0.2012	100.00%
Omitting Hustey et al. 2007	0.3671	[0.3340; 0.4008]	0.0405	0.2013	100.00%
Omitting Lund et al. 2011	0.3661	[0.3331; 0.3998]	0.0405	0.2012	100.00%
Omitting Maggiore et al. 2014-1	0.3672	[0.3341; 0.4010]	0.0405	0.2012	100.00%
Omitting Maggiore et al. 2014-2	0.369	[0.3362; 0.4024]	0.0398	0.1994	100.00%
Omitting Maio et al. 2006	0.3676	[0.3346; 0.4013]	0.0404	0.201	100.00%
Omitting Nightingale et al. 2022	0.3664	[0.3334; 0.4002]	0.0405	0.2013	100.00%
Omitting Pugh et al. 2005	0.3681	[0.3351; 0.4017]	0.0402	0.2006	100.00%
Omitting Pyszka et al. 2010	0.3651	[0.3322; 0.3987]	0.0402	0.2004	100.00%
Omitting Skaar et al. 2017	0.365	[0.3321; 0.3985]	0.0401	0.2003	100.00%
Omitting Viswanathan et al. 2005	0.3687	[0.3358; 0.4022]	0.0399	0.1998	100.00%
Omitting Weston et al. 2010	0.368	[0.3350; 0.4016]	0.0403	0.2007	100.00%
Omitting Baldoni et al. 2014-1	0.3658	[0.3328; 0.3995]	0.0404	0.2011	100.00%
Omitting Baldoni et al. 2014-2	0.365	[0.3321; 0.3985]	0.0401	0.2003	100.00%
Omitting da Costa et al. 2019	0.3636	[0.3311; 0.3967]	0.0392	0.1979	100.00%
Omitting Faustino et al. 2013	0.3674	[0.3344; 0.4012]	0.0404	0.2011	100.00%
Omitting Fulone et al. 2017-1	0.3672	[0.3341; 0.4009]	0.0405	0.2012	100.00%
Omitting Fulone et al. 2017-2	0.3664	[0.3334; 0.4002]	0.0405	0.2013	100.00%
Omitting Marques et al. 2018	0.3677	[0.3346; 0.4013]	0.0404	0.2009	100.00%
Omitting Oliveira et al. 2020	0.3638	[0.3313; 0.3970]	0.0394	0.1985	100.00%
Omitting Reis et al. 2017	0.3658	[0.3328; 0.3995]	0.0404	0.201	100.00%
Omitting Viana et al. 2022	0.3664	[0.3333; 0.4001]	0.0405	0.2013	100.00%
Omitting abubakar et al. 2021-1	0.3644	[0.3317; 0.3978]	0.0398	0.1995	100.00%
Omitting abubakar et al. 2021-2	0.3664	[0.3333; 0.4001]	0.0405	0.2013	100.00%
Omitting Akande-Sholabi et al. 2018	0.3671	[0.3340; 0.4008]	0.0405	0.2013	100.00%
Omitting Fadare et al. 2013	0.3675	[0.3345; 0.4013]	0.0404	0.201	100.00%
Omitting Saka et al. 2019-1	0.3668	[0.3337; 0.4005]	0.0405	0.2013	100.00%
Omitting Bhagavathula et al. 2021	0.3659	[0.3328; 0.3996]	0.0404	0.2011	100.00%
Omitting Lemma et al. 2020	0.3659	[0.3329; 0.3996]	0.0404	0.2011	100.00%
Omitting Tesfaye et al. 2020-1	0.3631	[0.3308; 0.3959]	0.0387	0.1966	100.00%
Omitting Tesfaye et al. 2020-2	0.366	[0.3330; 0.3997]	0.0405	0.2012	100.00%
Omitting Cross et al. 2016	0.3679	[0.3349; 0.4016]	0.0403	0.2008	100.00%
Omitting Saarelainen et al. 2014	0.3675	[0.3344; 0.4012]	0.0404	0.2011	100.00%
Pooled estimate	0.3667	[0.3338; 0.4001]	0.0402	0.2005	100.00%

© 2023 Tian F et al. *JAMA Network Open.*

eAppendix 9. Funnel plot for the prevalence of PIMs

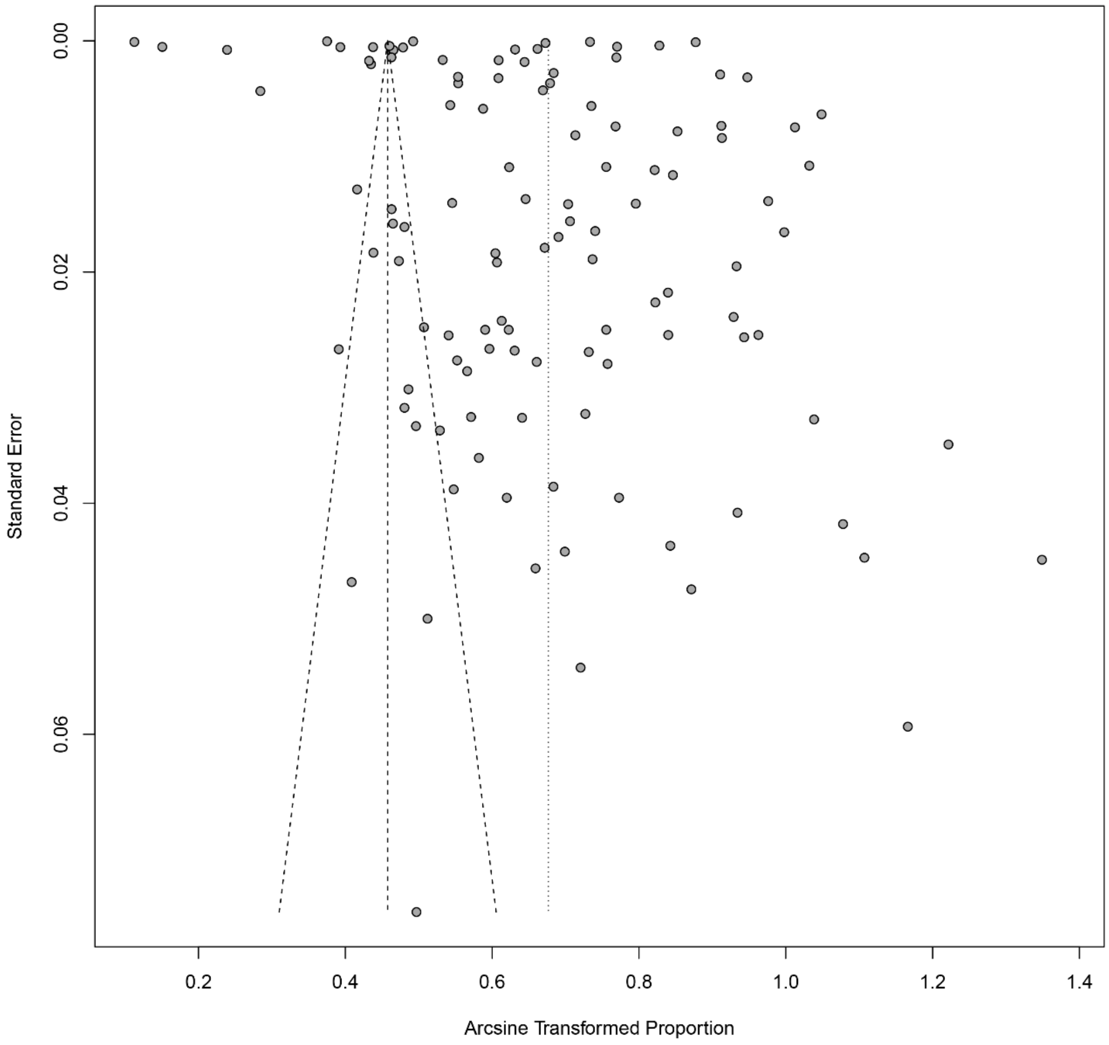

Egger's test:

```
> metabias(meta.data,method.bias="linreg")
Linear regression test of funnel plot asymmetry

Test result: t = 1.81, df = 130, p-value = 0.0732

Sample estimates:
     bias se.bias intercept se.intercept
 102.7158 56.8665    0.4436       0.0170
```

© 2023 Tian F et al. *JAMA Network Open.*

eReferences

[1] Chang CB, Lai HY, Hwang SJ, et al. The application of updating PIM-Taiwan criteria in clinic-visiting older patients with polypharmacy. Ther Adv Drug Saf. 2018 Oct 15;9(12):699-709. doi: 10.1177/2042098618804493.

[2] Chen Q, Zhang L. Analysis of potentially inappropriate medications (PIM) used in elderly outpatients in departments of internal medicine by using the Screening Tool of Older Persons' Potentially Inappropriate Prescriptions (STOPP) criteria. Ann Palliat Med. 2021 Apr;10(4):4678-4686. doi: 10.21037/apm-21-799.

[3] Huang Y, Zhang L, Huang X, et al. Potentially inappropriate medications in Chinese community-dwelling older adults. Int J Clin Pharm. 2020 Apr;42(2):598-603. doi: 10.1007/s11096-020-00980-y.

[4] Lin YJ, Peng LN, Chen LK, et al. Risk factors of potentially inappropriate medications among older patients visiting the community health center in rural Taiwan. Arch Gerontol Geriatr. 2011 Sep-Oct;53(2):225-8. doi: 10.1016/j.archger.2010.11.017.

[5] Lu L, Yao K, Chen J, Yang Y, et al. Prevalence of potentially inappropriate medications and association with comorbidities in older adults with diabetes in an outpatient visitation setting. Front Public Health. 2022 Sep 20;10:995948. doi: 10.3389/fpubh.2022.995948.

[6] Tian F, Li H, Chen Z, ea al. Potentially inappropriate medications in Chinese older outpatients in tertiary hospitals according to Beers criteria: A cross-sectional study. Int J Clin Pract. 2021 Aug;75(8):e14348. doi: 10.1111/ijcp.14348.

[7] Tian F, Zhao M, Chen Z, et al. Prescription of Potentially Inappropriate Medication Use in Older Cancer Outpatients With Multimorbidity: Concordance Among the Chinese, AGS/Beers, and STOPP Criteria. Front Pharmacol. 2022 Apr 12;13:857811. doi: 10.3389/fphar.2022.857811.

[8] Tian F, Chen Z, Tang R, et al. Impact of the expert consensus on polypharmacy and potentially inappropriate medication use in elderly lung cancer outpatients with multimorbidity: An interrupted time series analysis, 2016-2021. Front Pharmacol. 2022 Oct 5;13:992394. doi: 10.3389/fphar.2022.992394.

[9] Weng MC, Tsai CF, Sheu KL, et al. The impact of number of drugs prescribed on the risk of potentially inappropriate medication among outpatient older adults with chronic diseases. QJM. 2013 Nov;106(11):1009-15. doi: 10.1093/qjmed/hct141.

[10] Zeng Y, Yu Y, Liu Q, et al. Comparison of the prevalence and nature of potentially inappropriate medication use in geriatric outpatients between tertiary and community healthcare settings: a multicenter cross-sectional study. Int J Clin Pharm. 2022 Jun;44(3):619-629. doi: 10.1007/s11096-022-01380-0.

[11] Zhang H, Wong ELY, Wong SYS, et al. Comparison of adaptive versions of the Hong Kong-specific criteria and 2015 Beers criteria for assessing potentially inappropriate medication use in Hong Kong older patients. BMC Geriatr. 2021 Jun 21;21(1):379. doi: 10.1186/s12877-021-02324-5.

[12] Zhang Y, Chen Z, Tian F. Potentially inappropriate medications in older Chinese outpatients based on the Beers criteria and Chinese criteria. Front Pharmacol. 2022 Sep 30;13:991087. doi: 10.3389/fphar.2022.991087.

[13] Zhao M, Chen Z, Tian F, et al. Potentially Inappropriate Medication Among People With Dementia in China: A Nationwide Cross-Sectional Study. Front Pharmacol. 2022 Jun 13;13:929584. doi: 10.3389/fphar.2022.929584.

[14] Anand P, Katyal J, Dey AB, et al. Characterization of potentially inappropriate medications use in Indian elderly population and their impact on quality of life using Beers criteria. Aging Med (Milton). 2022 Feb 5;5(1):45-52. doi: 10.1002/agm2.12194.

[15] Bhatt AN, Paul SS, Krishnamoorthy S, et al, Mathew A, Nair BR. Potentially inappropriate medications prescribed for older persons: A study from two teaching hospitals in Southern India. J Family Community Med. 2019 Sep-Dec;26(3):187-192. doi: 10.4103/jfcm.JFCM_81_19.

[16] Mugada V, Bhagavathi KC, Sagina P, et al. Potentially inappropriate medications, drug-drug interactions, and prescribing practices in elderly patients: a cross-sectional study. Rev Assoc Med Bras (1992). 2021 Jun;67(6):800-805. doi: 10.1590/1806-9282.20200912.

[17] Shah KN, Joshi HM, Christian RP, et al. Prevalence of potentially inappropriate medications and prescription cost analysis among older cardiac patients in an outpatient department of a tertiary care hospital in India. J Basic Clin Pharm. 2016 Sep;7(4):110-115. doi: 10.4103/0976-0105.189434.
[18] Sharma R, Bansal P, Sharma A, et al. Prevalence and predictors of Potentially Inappropriate Psychotropic Medication in older adults with psychiatric illness. Asian J Psychiatr. 2021 Dec;66:102872. doi: 10.1016/j.ajp.2021.102872.
[19] Zaveri HG, Mansuri SM, Patel VJ. Use of potentially inappropriate medicines in elderly: A prospective study in medicine out-patient department of a tertiary care teaching hospital. Indian J Pharmacol. 2010 Apr;42(2):95-8. doi: 10.4103/0253-7613.64499.
[20] Patel MM, Mark AS, Shah NA. Assessment of appropriateness of prescribing among Indian geriatric outpatients using STOPP and START Criteria Version 2 at a tertiary care teaching hospital. Natl J Physiol Pharm Pharmacol. 2022 May; 12(10): 1746-1750. doi:10.5455/njppp.2023.13.05208202221052022
[21] Arai S, Ishikawa T, Kato H, et al. Multidrug use positively correlates with high-risk prescriptions in the Japanese elderly: a longitudinal study. J Pharm Health Care Sci. 2019 Sep 2;5:20. doi: 10.1186/s40780-019-0150-6.
[22] Bonfiglio V, Umegaki H, Kuzuya M. Potentially Inappropriate Medications and Polypharmacy: A Study of Older People with Mild Cognitive Impairment and Mild Dementia. J Alzheimers Dis. 2019;71(3):889-897. doi: 10.3233/JAD-190284.
[23] Fujie K, Kamei R, Araki R, et al. Prescription of potentially inappropriate medications in elderly outpatients: a survey using 2015 Japanese Guidelines. Int J Clin Pharm. 2020 Apr;42(2):579-587. doi: 10.1007/s11096-020-00967-9. Epub 2020 Jan 21.
[24] Masumoto S, Sato M, Maeno T, et al. Association between potentially inappropriate medications and anxiety in Japanese older patients. Geriatr Gerontol Int. 2017 Dec;17(12):2520-2526. doi: 10.1111/ggi.13128.
[25] Suzuki Y, Shiraishi N, Komiya H, et al. Potentially inappropriate medications increase while prevalence of polypharmacy/hyperpolypharmacy decreases in Japan: A comparison of nationwide prescribing data. Arch Gerontol Geriatr. 2022 Sep-Oct;102:104733. doi: 10.1016/j.archger.2022.104733.
[26] Uragami Y, Takikawa K, Kareki H, et al. Effect of number of medications and use of potentially inappropriate medications on frailty among early-stage older outpatients. J Pharm Health Care Sci. 2021 May 3;7(1):15. doi: 10.1186/s40780-021-00195-x.
[27] Bahat G, Bay I, Tufan A, et al. Prevalence of potentially inappropriate prescribing among older adults: A comparison of the Beers 2012 and Screening Tool of Older Person's Prescriptions criteria version 2. Geriatr Gerontol Int. 2017 Sep;17(9):1245-1251. doi: 10.1111/ggi.12850.
[28] Kelleci Çakır B, Kızılarslanoğlu MC, Kılıç MK, et al. Assessment of the Appropriateness of Prescriptions in a Geriatric Outpatient Clinic. Turk J Pharm Sci. 2022 Feb 28;19(1):54-62. doi: 10.4274/tjps.galenos.2021.77010.
[29] Paksoy C, Özkan Ö, Ustaalioğlu BB, et al. Evaluation of potentially inappropriate medication utilization in elderly patients with cancer at outpatient oncology unit. J Oncol Pharm Pract. 2019 Sep;25(6):1321-1327. doi: 10.1177/1078155218788698.
[30] Pala E, Ersoy S, Engin VS, et al. Effectiveness of STOPP/START criteria in primary prevention of polypharmacy and under-treatment in older patients. Therapie. 2022 May-Jun;77(3):361-369. doi: 10.1016/j.therap.2021.07.003.
[31] ŞAHİN E K, HAMURCU H D, ÇAYKÖYLÜ A, et al. Evaluation of the potentially inappropriate psychotropic use in elderly psychiatric patients[J]. Turkish Journal of Geriatrics/Türk Geriatri Dergisi; 2021 Sep; 24(4).
[32] Sari YE, Unluoglu I, Cavusoglu Y, et al. Inappropriate drug use rates of geriatric patients attending to a university hospital cardiology policlinic. Niger J Clin Pract. 2021 Nov;24(11):1719-1727. doi: 10.4103/njcp.njcp_517_18.
[33] Cho ST, Kim JS, Noh J, et al. Characteristics of inappropriate multiple medication use in older urological outpatients. Arch Gerontol Geriatr. 2019 Jul-Aug;83:61-65. doi:

10.1016/j.archger.2019.03.024.
[34] Kim DS, Huh S, Lee S. Potentially inappropriate medication use at ambulatory care visits by elderly patients covered by National Health Insurance in Korea. Int J Clin Pharmacol Ther. 2015 Oct;53(10):819-27. doi: 10.5414/CP202429.
[35] Kim J, Han E, Hwang HJ, et al. Potentially inappropriate prescribing of cardiovascular system and antiplatelet/anticoagulant drugs among elderly patients: a Korean population-based national study. J Geriatr Cardiol. 2021 May 28;18(5):327-337. doi: 10.11909/j.issn.1671-5411.2021.05.010.
[36] Lim YJ, Kim HY, Choi J, et al. Potentially Inappropriate Medications by Beers Criteria in Older Outpatients: Prevalence and Risk Factors. Korean J Fam Med. 2016 Nov;37(6):329-333. doi: 10.4082/kjfm.2016.37.6.329. Epub 2016 Nov 18.
[37] Yoon K, Kim JT, Kwack WG, et al. Potentially Inappropriate Medication Use in Patients with Dementia. Int J Environ Res Public Health. 2022 Sep 10;19(18):11426. doi: 10.3390/ijerph191811426.
[38] Al-Omar HA, Al-Sultan MS, Abu-Auda HS. Prescribing of potentially inappropriate medications among the elderly population in an ambulatory care setting in a Saudi military hospital: trend and cost. Geriatr Gerontol Int. 2013 Jul;13(3):616-21. doi: 10.1111/j.1447-0594.2012.00951.x.
[39] Alwhaibi M. Potentially Inappropriate Medications Use among Older Adults with Comorbid Diabetes and Hypertension in an Ambulatory Care Setting. J Diabetes Res. 2022 May 9;2022:1591511. doi: 10.1155/2022/1591511.
[40] Alhawassi TM, Alatawi W, Alwhaibi M. Prevalence of potentially inappropriate medications use among older adults and risk factors using the 2015 American Geriatrics Society Beers criteria. BMC Geriatr. 2019 May 29;19(1):154. doi: 10.1186/s12877-019-1168-1.
[41] Meraya AM, Banji OJF, Khobrani MA, et al. Evaluation of psychotropic medications use among elderly with psychiatric disorders in Saudi Arabia. Saudi Pharm J. 2021 Jun;29(6):603-608. doi: 10.1016/j.jsps.2021.04.021.
[42] Cojutti P, Arnoldo L, Cattani G, et al. Polytherapy and the risk of potentially inappropriate prescriptions (PIPs) among elderly and very elderly patients in three different settings (hospital, community, long-term care facilities) of the Friuli Venezia Giulia region, Italy: are the very elderly at higher risk of PIPs? Pharmacoepidemiol Drug Saf. 2016 Sep;25(9):1070-8. doi: 10.1002/pds.4026.
[43] Galimberti F, Casula M, Scotti L, et al. Potentially Inappropriate Prescribing among Elderly Outpatients: Evaluation of Temporal Trends 2012-2018 in Piedmont, Italy. Int J Environ Res Public Health. 2022 Mar 18;19(6):3612. doi: 10.3390/ijerph19063612.
[44] Loddo S, Salis F, Rundeddu S, et al. Nutritional Status and Potentially Inappropriate Medications in Elderly. J Clin Med. 2022 Jun 16;11(12):3465. doi: 10.3390/jcm11123465.
[45] Maio V, Yuen EJ, Novielli K, et al. Potentially inappropriate medication prescribing for elderly outpatients in Emilia Romagna, Italy: a population-based cohort study. Drugs Aging. 2006;23(11):915-24. doi: 10.2165/00002512-200623110-00006.
[46] Maio V, Del Canale S, Abouzaid S; GAP Investigators. Using explicit criteria to evaluate the quality of prescribing in elderly Italian outpatients: a cohort study. J Clin Pharm Ther. 2010 Apr;35(2):219-29. doi: 10.1111/j.1365-2710.2009.01094.x.
[47] Varga S, Alcusky M, Keith SW, Hegarty SE, Del Canale S, Lombardi M, Maio V. Hospitalization rates during potentially inappropriate medication use in a large population-based cohort of older adults. Br J Clin Pharmacol. 2017 Nov;83(11):2572-2580. doi: 10.1111/bcp.13365.
[48] Bongue B, Naudin F, Laroche ML, et al. Trends of the potentially inappropriate medication consumption over 10 years in older adults in the East of France. Pharmacoepidemiol Drug Saf. 2009 Dec;18(12):1125-33. doi: 10.1002/pds.1762.
[49] Choukroun C, Leguelinel-Blache G, Roux-Marson C, et al. Impact of a pharmacist and geriatrician medication review on drug-related problems in older outpatients with cancer. J Geriatr Oncol. 2021 Jan;12(1):57-63. doi: 10.1016/j.jgo.2020.07.010.
[50] Drusch S, Le Tri T, Ankri J, et al. Decreasing trends in potentially inappropriate medications in older people: a nationwide repeated cross-sectional study. BMC Geriatr. 2021 Nov 2;21(1):621. doi: 10.1186/s12877-021-02568-1.

© 2023 Tian F et al. *JAMA Network Open.*

[51] Dubois-Puechlong S, Mille F, Hindlet P, et al. Potentially inappropriate prescriptions of antithrombotic therapies in older outpatients: a French multicenter cross-sectional study. Eur Geriatr Med. 2019 Jun;10(3):473-481. doi: 10.1007/s41999-019-00176-2.
[52] Dormann H, Sonst A, Müller F, et al. Adverse drug events in older patients admitted as an emergency: the role of potentially inappropriate medication in elderly people (PRISCUS). Dtsch Arztebl Int. 2013 Mar;110(13):213-9. doi: 10.3238/arztebl.2013.0213.
[53] Endres HG, Kaufmann-Kolle P, Steeb V, et al. Association between Potentially Inappropriate Medication (PIM) Use and Risk of Hospitalization in Older Adults: An Observational Study Based on Routine Data Comparing PIM Use with Use of PIM Alternatives. PLoS One. 2016 Feb 3;11(2):e0146811. doi: 10.1371/journal.pone.0146811.
[54] Fiss T, Dreier A, Meinke C, et al. Frequency of inappropriate drugs in primary care: analysis of a sample of immobile patients who received periodic home visits. Age Ageing. 2011 Jan;40(1):66-73. doi: 10.1093/ageing/afq106.
[55] Gudd K, Meier F, Lindenthal J, et al. Potenziell inadäquate Medikation in einem deutschen Praxisnetz – Wer verordnet was an wen? [Potentially inappropriate medication in a German practice network-who prescribes what to whom?]. Z Gerontol Geriatr. 2020 Nov;53(7):647-654. German. doi: 10.1007/s00391-019-01660-x.
[56] Ham L, Geijteman ECT, Aarts MJ, et al. Use of potentially inappropriate medication in older patients with lung cancer at the end of life. J Geriatr Oncol. 2022 Jan;13(1):53-59. doi: 10.1016/j.jgo.2021.07.009.
[57] Boersma MN, Huibers CJA, Drenth-van Maanen AC, et al. The effect of providing prescribing recommendations on appropriate prescribing: A cluster-randomized controlled trial in older adults in a preoperative setting. Br J Clin Pharmacol. 2019 Sep;85(9):1974-1983. doi: 10.1111/bcp.13987.
[58] Oktora MP, Alfian SD, Bos HJ, et al. Trends in polypharmacy and potentially inappropriate medication (PIM) in older and middle-aged people treated for diabetes. Br J Clin Pharmacol. 2021 Jul;87(7):2807-2817. doi: 10.1111/bcp.14685.
[59] van Loveren FMAM, van Berlo-van de Laar IRF, Imholz ALT, et al. Prevalence and follow-up of potentially inappropriate medication and potentially omitted medication in older patients with cancer - The PIM POM study. J Geriatr Oncol. 2021 Jan;12(1):80-84. doi: 10.1016/j.jgo.2020.06.014.
[60] Santos-Pérez MI, Fierro I, Salgueiro-Vázquez ME, et al. A cross-sectional study of psychotropic drug use in the elderly: Consuming patterns, risk factors and potentially inappropriate use. Eur J Hosp Pharm. 2021 Mar;28(2):88-93. doi: 10.1136/ejhpharm-2019-001927.
[61] Yeste-Gómez I, Durán-García ME, Muiño-Miguez A, et al. Prescripciones potencialmente inapropiadas en el tratamiento ambulatorio de pacientes ancianos [Potentially inappropriate prescriptions in the ambulatory treatment of elderly patients]. Rev Calid Asist. 2014 Jan-Feb;29(1):22-8. Spanish. doi: 10.1016/j.cali.2013.08.001.
[62] Cvetković Z, Perić A, Dobrić S. Potentially Inappropriate Prescribing and Potential Clinically Significant Drug-Drug Interactions in Older Outpatients: Is There Any Association? Medicina (Kaunas). 2019 Jul 3;55(7):332. doi: 10.3390/medicina55070332.
[63] Projovic I, Vukadinovic D, Milovanovic O, et al. Risk factors for potentially inappropriate prescribing to older patients in primary care. Eur J Clin Pharmacol. 2016 Jan;72(1):93-107. doi: 10.1007/s00228-015-1957-1.
[64] Buck MD, Atreja A, Brunker CP, et al. Potentially inappropriate medication prescribing in outpatient practices: prevalence and patient characteristics based on electronic health records. Am J Geriatr Pharmacother. 2009 Apr;7(2):84-92. doi: 10.1016/j.amjopharm.2009.03.001.
[65] Curtis LH, Østbye T, Sendersky V, et al. Inappropriate prescribing for elderly Americans in a large outpatient population. Arch Intern Med. 2004 Aug 9-23;164(15):1621-5. doi: 10.1001/archinte.164.15.1621.
[66] Dunn RL, Harrison D, Ripley TL. The beers criteria as an outpatient screening tool for potentially inappropriate medications. Consult Pharm. 2011 Oct;26(10):754-63. doi: 10.4140/TCP.n.2011.754.
[67] Gardner K, Schwarz K, Pearson S, et al. Potentially Inappropriate Medication Usage in Older Adults in a Tertiary Academic Medical Center Emergency Department. J Pharm Pract. 2022

Dec;35(6):892-897. doi: 10.1177/08971900211016813.
[68] Hustey FM, Wallis N, Miller J. Inappropriate prescribing in an older ED population. Am J Emerg Med. 2007 Sep;25(7):804-7. doi: 10.1016/j.ajem.2007.01.018.
[69] Lund BC, Steinman MA, Chrischilles EA, et al. Beers criteria as a proxy for inappropriate prescribing of other medications among older adults. Ann Pharmacother. 2011 Nov;45(11):1363-70. doi: 10.1345/aph.1Q361.
[70] Maggiore RJ, Dale W, Gross CP, et al. Polypharmacy and potentially inappropriate medication use in older adults with cancer undergoing chemotherapy: effect on chemotherapy-related toxicity and hospitalization during treatment. J Am Geriatr Soc. 2014 Aug;62(8):1505-12. doi: 10.1111/jgs.12942.
[71] Maio V, Hartmann CW, Poston S, et al. Potentially inappropriate prescribing for elderly patients in 2 outpatient settings. Am J Med Qual. 2006 May-Jun;21(3):162-8. doi: 10.1177/1062860605285475.
[72] Nightingale G, Scopelliti EM, Casten R, et al. Polypharmacy and Potentially Inappropriate Medication Use in Older Blacks with Diabetes Mellitus Presenting to the Emergency Department. J Aging Health. 2022 Aug-Sep;34(4-5):499-507. doi: 10.1177/08982643211045546. Epub 2021 Sep 13.
[73] Pugh MJ, Fincke BG, Bierman AS, et al. Potentially inappropriate prescribing in elderly veterans: are we using the wrong drug, wrong dose, or wrong duration? J Am Geriatr Soc. 2005 Aug;53(8):1282-9. doi: 10.1111/j.1532-5415.2005.53402.x.
[74] Pyszka LL, Seys Ranola TM, Milhans SM. Identification of inappropriate prescribing in geriatrics at a Veterans Affairs hospital using STOPP/START screening tools. Consult Pharm. 2010 Jun;25(6):365-73. doi: 10.4140/TCP.n.2010.365.
[75] Skaar DD, O'Connor H. Using the Beers criteria to identify potentially inappropriate medication use by older adult dental patients. J Am Dent Assoc. 2017 May;148(5):298-307. doi: 10.1016/j.adaj.2017.01.020.
[76] Viswanathan H, Bharmal M, Thomas J 3rd. Prevalence and correlates of potentially inappropriate prescribing among ambulatory older patients in the year 2001: comparison of three explicit criteria. Clin Ther. 2005 Jan;27(1):88-99. doi: 10.1016/j.clinthera.2005.01.009.
[77] Weston AL, Weinstein AM, Barton C, et al. Potentially inappropriate medication use in older adults with mild cognitive impairment. J Gerontol A Biol Sci Med Sci. 2010 Mar;65(3):318-21. doi: 10.1093/gerona/glp158.
[78] Baldoni Ade O, Ayres LR, Martinez EZ, et al. Factors associated with potentially inappropriate medications use by the elderly according to Beers criteria 2003 and 2012. Int J Clin Pharm. 2014 Apr;36(2):316-24. doi: 10.1007/s11096-013-9880-y.
[79] da Costa IHF, Silva RME, Carlos JO, et al. Potentially inappropriate medications in older kidney transplant recipients: a Brazilian prevalence study. Int J Clin Pharm. 2019 Aug;41(4):888-894. doi: 10.1007/s11096-019-00842-2.
[80] Faustino CG, Passarelli MC, Jacob-Filho W. Potentially inappropriate medications among elderly Brazilian outpatients. Sao Paulo Med J. 2013;131(1):19-26. doi: 10.1590/s1516-31802013000100004.
[81] Fulone I, Lopes LC. Potentially inappropriate prescriptions for elderly people taking antidepressant: comparative tools. BMC Geriatr. 2017 Dec 2;17(1):278. doi: 10.1186/s12877-017-0674-2.
[82] Marques GFM, Rezende DMRP, Silva IPD, et al. Polypharmacy and potentially inappropriate medications for elder people in gerontological nursing. Rev Bras Enferm. 2018 Sep-Oct;71(5):2440-2446. English, Portuguese. doi: 10.1590/0034-7167-2017-0211.
[83] Oliveira HSB, Michels AC, Nahime M, et al. Risk factors associated with potential cardiovascular and cerebrovascular adverse events in elderly individuals assisted at secondary level. Rev Assoc Med Bras (1992). 2020 Aug;66(8):1087-1092. doi: 10.1590/1806-9282.66.8.1087.
[84] Reis CM, Dos Santos AG, de Jesus Souza P, et al. Factors associated with the use of potentially inappropriate medications by older adults with cancer. J Geriatr Oncol. 2017 Jul;8(4):303-307. doi: 10.1016/j.jgo.2017.05.003.

[85] Viana SSC, Souza NPDS, Aliberti MJR, et al. Use of potentially inappropriate medications and adverse events in older outpatients with acute conditions. Einstein (Sao Paulo). 2022 Jul 4;20:eAO8024. doi: 10.31744/einstein_journal/2022AO8024.
[86] Abubakar U, Tangiisuran B, Kolo M, et al. Prevalence and predictors of potentially inappropriate medication use among ambulatory older adults in Northern Nigeria[J]. Drugs & Therapy Perspectives, 2021, 37: 94-99.
[87] Akande-Sholabi W, Adebusoye LA, Olowookere OO. Potentially inappropriate medication use among older patients attending a geriatric centre in south-west Nigeria. Pharm Pract (Granada). 2018 Jul-Sep;16(3):1235. doi: 10.18549/PharmPract.2018.03.1235.
[88] Fadare JO, Agboola SM, Opeke OA, et al. Prescription pattern and prevalence of potentially inappropriate medications among elderly patients in a Nigerian rural tertiary hospital. Ther Clin Risk Manag. 2013;9:115-20. doi: 10.2147/TCRM.S40120.
[89] Saka SA, Oosthuizen F, Nlooto M. Potential inappropriate prescribing and associated factors among older persons in Nigeria and South Africa. Int J Clin Pharm. 2019 Feb;41(1):207-214. doi: 10.1007/s11096-018-0770-1.
[90] Bhagavathula AS, Seid MA, Adane A, et al. Prevalence and Determinants of Multimorbidity, Polypharmacy, and Potentially Inappropriate Medication Use in the Older Outpatients: Findings from EuroAgeism H2020 ESR7 Project in Ethiopia. Pharmaceuticals (Basel). 2021 Aug 25;14(9):844. doi: 10.3390/ph14090844.
[91] Lemma W, Islam M, Loha E. Potentially inappropriate medication prescribing patterns in geriatric patients in a health facility in Addis Ababa, Ethiopia[J]. Tropical Journal of Pharmaceutical Research, 2020, 19(11): 2441-2446.
[92] Tesfaye BT, Tessema MT, Yizengaw MA, et al. Potentially inappropriate medication use among older adult patients on follow-up at the chronic care clinic of a specialized teaching hospital in Ethiopia. A cross-sectional study. BMC Geriatr. 2021 Oct 7;21(1):530. doi: 10.1186/s12877-021-02463-9.
[93] Cross AJ, George J, Woodward MC, et al. Potentially Inappropriate Medications and Anticholinergic Burden in Older People Attending Memory Clinics in Australia. Drugs Aging. 2016 Jan;33(1):37-44. doi: 10.1007/s40266-015-0332-3.
[94] Saarelainen LK, Turner JP, Shakib S, et al. Potentially inappropriate medication use in older people with cancer: prevalence and correlates. J Geriatr Oncol. 2014 Oct 1;5(4):439-46. doi: 10.1016/j.jgo.2014.07.001.
