# Supplementary material for: Prevalence of Use of Potentially Inappropriate Medications Among Older Adults Worldwide: A Systematic Review and Meta-Analysis
Source: JAMA Netw Open. 2023 Aug 2;6(8):e2326910. doi: 10.1001/jamanetworkopen.2023.26910 (PMC10398411; doi:10.1001/jamanetworkopen.2023.26910)
Supplement: Supplement 2. — Data Sharing Statement [file jamanetwopen-e2326910-s002.pdf]

Data Sharing Statement

Tian. Prevalence of Use of Potentially Inappropriate Medications Among Older Adults Worldwide. *JAMA Netw Open*. Published August 02, 2023.
doi:10.1001/jamanetworkopen.2023.26910

Data
Data available: No
